# Knockout of eight *hydroxyproline-O-galactosyltransferases* cause multiple vegetative and reproductive growth defects

**DOI:** 10.1016/j.tcsw.2023.100117

**Published:** 2023-11-25

**Authors:** Dasmeet Kaur, Michael A. Held, Yuan Zhang, Diana Moreira, Silvia Coimbra, Allan M. Showalter

**Affiliations:** a Department of Environmental & Plant Biology, Ohio University, Athens, OH 45701-2979, USA; b Molecular and Cellular Biology Program, Ohio University, Athens, OH 45701-2979, USA; c Department of Chemistry and Biochemistry, Ohio University, Athens, OH 45701-2979, USA; d LAQV/REQUIMTE, Departamento de Biologia, Faculdade de Ciências, Universidade do Porto, Rua do Campo Alegre s/n, 4169-007, Porto, Portugal

**Keywords:** Arabinogalactan-proteins, Hydroxyproline-galactosyltransferases, Plant growth, Reproduction, Stamen defects, Pollen development, Pollen grain

## Abstract

Arabinogalactan-proteins (AGPs) are a family of hyperglycosylated hydroxyproline-rich cell wall proteins found throughout the plant kingdom. To date, eight Hydroxyproline-galactosyltransferases (Hyp-GALTs), named GALT2-GALT9, are known to catalyze the addition of the first galactose sugar to Hyp residues in AGP protein cores. The generation and characterization of *galt23456789* octuple mutants using CRISPR-Cas9 gene editing technology, provided strong reverse genetic evidence that AG glycans are essential for normal vegetative and reproductive growth, as these mutants demonstrated stunted growth, greatly delayed flowering and significant defects in floral organ development and morphogenesis. Compared to the lower seed set of *galt25789* quintuple mutants being more so contributed by female gametophytic defects, dramatically low seed-set of octuple mutants was largely due to impaired male reproductive function, specifically due to shorter filaments, delayed anther dehiscence, and large decreases in pollen quantity and viability. Octuple mutant pollen had severely distorted reticulate exine, tectum patterning and intine thickness. Reduced amounts of galactose and arabinose in overall lower amounts of β-Yariv precipitated AGPs illustrated how biological functions of AGPs are affected by abnormal glycosylation.

## Highlights

• Eight Hyp-GALT genes are known to add galactose to arabinogalactan-protein cores.
• Mutation of eight Hyp-GALTs interrupts both vegetative and reproductive organ growth.
• Distorted pollen cell wall structures make them to collapse.
• Semi-sterile anthers and short filaments leads to extremely low seed-set.

## 1 Introduction

Arabinogalactan-proteins (AGPs) are a family of hydroxyproline (Hyp)-rich cell wall glycoproteins found throughout the plant kingdom. Hyp residues in AGP protein backbones are post-translationally *O*-glycosylated with type II arabinogalactan (AG) chains, which are rich in arabinose and galactose and added by the action of several AGP glycosyltransferases (GTs). In general, AGPs are composed of 2–10 % protein and 90–98 % carbohydrate, by weight (Majewska-Sawka and Nothnagel, 2000, Showalter, 2001). Thus, AG chains represent the bulk of most AGPs and largely constitute their interactive molecular surface. More than half of the AGPs contain a C-terminal glycosylphosphatidylinositol (GPI) anchor sequence, which tethers these AGPs to the outer leaflet of the plasma membrane (Borner et al., 2003). AGPs, and particularly their AG chains, are widely regarded as potential cell wall/plasma membrane signaling molecules (Silva et al., 2020).

Although AGPs account for less than 10 % of the plant cell wall, they are widely reported to control various aspects of plant growth and development. AGPs are implicated to function in cell expansion, cell division, programmed cell death, root formation and development, xylem differentiation, organ differentiation, male and female gametogenesis, interaction between stigma and pollen, pollen tube growth, communication between the pollen tube and pistil tissues until zygotic divisions and embryo development (Willats and Knox, 1996, Majewska-Sawka and Nothnagel, 2000, Seifert and Roberts, 2007, Okuda et al., 2013, Dresselhaus and Coimbra, 2016, Mizukami et al., 2016, Seifert, 2020). Moreover, certain AGPs show developmental and spatiotemporal expression patterns in various plant organs, including reproductive tissues.

In Arabidopsis, about 85 AGPs have been predicted (Showalter et al., 2010) and several AGP mutants have been characterized and display various growth defects. Based on the presence/absence of specific structural motifs/domains of AGP protein backbones, AGP subfamilies can be classified as: (i) classical AGPs, which contains a PAST-rich (Pro, Arg, Ser, Thr) region of variable length, along with a GPI-anchored signal; (ii) Lys-rich classical AGPs, which are a subclass of classical AGPs, their PAST regions include a short Lys-rich region; (iii) AG-peptides, which represent a subclass of classical AGPs of short length (<90amino acid residues); (iv) chimeric AGPs if there is a specific domain(s) in addition to the PAST-rich region such as Fasciclin-like AGPs (FLAs), phytocyanin-like AGPs (PAGs), and xylogen-like AGPs (XYLPs) and; (v) AGP hybrids, which have characteristics of AGPs and another HRGP sequence, such as extensins. Interestingly, the answer to the question of whether these eight Hyp-GALT enzymes act on all AGPs or on a subset of AGPs in specific organs at a given time remains unknown. AGP14, AGP29, FLA1, FLA4/SOS5, AGP17, AGP19, AGP30, and AGP31 are known to be involved in vegetative growth and development, including but not limited to processes like root growth, root hair elongation, lateral root initiation, root and shoot regeneration, seed germination, cell division and expansion (Gaspar et al., 2004, Yang et al., 2007, Johnson et al., 2011, Hijazi et al., 2012, Seifert et al., 2014, Showalter and Basu, 2016, Zhou, 2019). Additionally, several AGPs are involved in reproductive roles; for example, AGP6 and AGP11 in pollen viability along with precocious as well as reduced pollen tube growth (Coimbra et al., 2009, Coimbra and Pereira, 2012), AGP6, AGP11, AGP23, and AGP40 in pollen development (Nguema-Ona et al., 2012), AGP6 for nexine formation (Jia et al., 2015), FLA3 and FLA14 for pollen intine wall development (Li et al., 2010, Miao et al., 2021), AGP4/JAGGER for polytubey block coupled with degeneration of persistent synergids (Pereira et al., 2016), AGP18 in female gametogenesis (Acosta-García and Vielle-Calzada, 2004, Zhang et al., 2011), and early nodulin-like proteins (ENODL)11–15 in pollen tube reception by the ovule (Hou et al., 2016).

Angiosperms, by far the most successful and largest plant group, have a complex gametophytic development with distinct and spatially separated male and female haploid gametophytes contained in specialized sporophytic reproductive organs of the flower. Anthers, the male sporophytic reproductive organs, bear multiple pollen mother cells (PMC)/microsporocytes which undergo meiosis and mitosis events to develop into pollen grains, the male gametophytes enclosing sperm cells, the male gametes. Ovules, the female sporophytic reproductive organs, contain a single megaspore mother cell (MMC) that undergoes meiosis and mitosis events resulting in a seven celled female gametophyte, the embryo sac, which includes an egg cell and two synergids at the micropyle, two polar nuclei in the central cell and three antipodal cells at the chalaza pole of the embryo sac (Goldberg et al., 1994, Bleckmann et al., 2014). To initiate fertilization, the mature pollen grains stick to stigmatic papillae to hydrate, the pollen tubes emerge and penetrate competently through the nutrient-rich diploid transmitting tissues of the pistil to reach the female embryo sac in each ovule. The two immotile sperm cells will fertilize the two female gametes, the egg cell and the fused polar nuclei of the central cell, to initiate embryo and endosperm proliferation respectively (Nagahara et al., 2021). In *Torenia fournieri* ethyl-glucuronosyl galactose disaccharide molecules, likely originating from AGPs, prime the pollen tubes for recognition of the attractant LURE peptides, which are cysteine-rich peptides secreted from the two synergid cells and involved in pollen tube attraction (Takeuchi and Higashiyama, 2016, Dresselhaus and Coimbra, 2016, Jiao et al., 2017). An intricate network of largely undefined signaling events including growth stimulation, mechanical guidance, chemo-attraction, adhesion, reorientation, and competence control, appears to occur within and between male and female counterparts in this complex fertilization process (Johnson and Lord, 2006, Higashiyama and Hamamura, 2008, Bleckmann et al., 2014, Mizuta and Higashiyama, 2018). Cell walls, the dynamic structural entities largely composed of cellulosic and pectate molecules along with a diverse set of cell wall proteins, potentially mediate such cell–cell interactions.

Arabidopsis pollen grain development provides an ideal model system to study the role of Hyp-*O*-glycosides in pollen cell walls. The multilayered pollen wall is composed of a typical inner pectocellulosic intine and an outer exine made of sporopollenin (Grienenberger and Quilichini, 2021). The exine can be further subdivided into two layers, the inner nexine and outer sexine. The three-dimensional homogenous latticework of sexine reticulate architecture is precisely laid out by its elements: baculae rising like columns and tecta forming the roofs on these columns (Suzuki et al., 2017). The sporophytic tapetum in anther locules is thought to be involved in the formation of sporopollenin precursors regulating exine synthesis jointly with the male gametophyte (Zhou et al., 2015, Wang et al., 2018). Later, the pollen coat derived from the enzymatic catalysis of the tapetum is deposited over the exine cavities of the outer exine layer (Ariizumi and Steber, 2007, Liu and Fan, 2013, Quilichini et al., 2015). Both tapetal differentiation and tapetal degeneration by programmed cell death are critical for pollen-wall formation. Unlike the exine, intine synthesis appears to be controlled by the microspore itself (Jia et al., 2015). A mutation in the *KNS4/UPEX1* gene, which encodes a β-(1,3)-galactosyltransferase (GT31 family) for AGPs, results in abnormal primexine development (Suzuki et al., 2017). Furthermore, mutants of the *GLCAT14A/B/C* (GT14 family) genes, which encode glucuronic acid (GlcA) transferases for AGPs, results in intine and exine defects along with delayed embryo development (Zhang et al., 2020, Ajayi et al., 2021).

Eight hydroxyproline-galactosyltransferases (*Hyp-GALTs*) were characterized to date and act to add the first galactose (Gal) sugar to Hyp residues within AGP protein backbones (Basu et al., 2015a, Basu et al., 2015b, Ogawa-Ohnishi and Matsubayashi, 2015). Five of these eight Hyp-GALTs contain a galectin domain (GALT2-6 found in Clade 7, sub-clades V and VI of the GT31 family) in addition to a GALT domain, while the other three contain only a GALT domain (GALT7-9 found in Clade 10, subclade III of the GT31 family) (Qu et al., 2008). Corresponding to the wide distribution of AGP expression patterns, *Hyp-GALT* genes are also predicted to be expressed in various vegetative plant organs/tissues. Recently, we demonstrated fertilization failure coupled with male and female gametophytic defects in *galt2galt5galt7galt8galt9* quintuple mutants (Kaur et al., 2022, Moreira et al., 2023). Here, we extend that work and examine the roles of all eight *Hyp-GALT*s, using a reverse genetics approach, that combines both T-DNA mutants and CRISPR-Cas9 gene edited mutants. Specifically, we report here on the generation and phenotypic characterization of *galt2galt3galt4galt5galt6galt7galt8galt9* (*galt23456789*) octuple mutants with respect to their functional roles in vegetative and male reproductive growth and development in Arabidopsis.

## 2 Material and methods

### 2.1 Plant materials and growth conditions

*Arabidopsis thaliana* (Columbia-0 ecotype) was obtained from the Arabidopsis Biological Research Center (ABRC), Columbus, Ohio, USA and used as WT. Previously, the homozygous quintuple mutant *galt25789* was generated using conventional breeding of T-DNA mutants and *galt23456* was generated by using a CRISPR-Cas9 multiplexing approach (Kaur et al., 2021, Zhang et al., 2021). We generated the *galt23456789* octuple mutants in this study in a *galt25789* T-DNA mutant background using a CRISPR-Cas9 gene multiplexing approach. The genetic construct carrying four gRNAs designed to target *GALT3*, *GALT4*, and *GALT6* along with the vector construction have been described previously (Zhang et al., 2021). For this study, Agrobacterium strain GV3101 containing this construct cloned into the pHEE401E vector was used to transform Arabidopsis *galt25789* mutants via the floral dip method (Clough and Bent, 1998). Indel mutations were detected by Sanger sequencing and potential off target mutations were also checked by Sanger sequencing. All plants used in this study were germinated after 4 d of stratification in the dark at 4 °C and were grown in soil under long-day conditions (16 h of light/8h of dark, 22 °C, 60–70 % relative humidity) in growth chambers at a light intensity of 122 μmol m^−2^ s^−1^.

### 2.2 Seedling and aerial plant growth phenotyping

Plants were grown in soil for mutant screening, and growth-stage phenotypic analysis as described by Kaur et al. (2021). Plant height was measured at 45 d after germination under normal conditions (*n* > 50) in three independent experiments. Flowering time was also recorded. For monitoring root growth in response to β-Gal-Yariv reagent, all seedlings were grown on ½ MS plates for 4 d and were then transferred to ½ MS supplemented with 50 μM β-Gal-Yariv reagent. Root lengths were measured using Image J software.

### 2.3 Monosaccharide composition analysis by HPAEC-PAD

AGPs were isolated from Arabidopsis siliques and inflorescences of WT and *Hyp-GALT* mutants following methods described previously (Lamport, 2013) and were subjected to high pH anion-exchange chromatography with pulsed amperometric detection (HPAEC-PAD) using a Dionex PA-20 column (Thermo Fisher Scientific, Sunnyvale, CA, USA) as described earlier (Kaur et al., 2021). Briefly, AGPs were extracted from siliques and inflorescences of 45–50-day-old (∼8–10 g of silique) WT and Hyp-GALT mutant plants. The tissues which were snap-frozen with liquid nitrogen, ground to a fine powder and mixed with 2 % NaCl (1 part tissue: 4 parts of 2 % NaCl), followed by shaking at 200 rpm for 3 h at room temperature. Samples were centrifuged at 13,000 g for 30 min and the supernatant obtained was treated with 2 ml of β-D-Gal-Yariv reagent dissolved in 2 % NaCl (2 mg/mL) overnight. The precipitated AGPs were collected by centrifugation for 20 min at 2000 g, washed with 2 % NaCl twice, resuspended in 2 ml H_2_O. Sodium dithionite was added and incubated for 15 min at 50 °C until the mixture was decolorized. The resulting solution was desalted on a PD-10 column (GE Healthcare) that had been equilibrated with water, and the eluate was freeze-dried overnight. Then, monosaccharide composition analysis was done with approximately 500 μg of AGPs, which were first hydrolyzed using 2 N TFA, at 121 °C for 90 min followed by removal of TFA by drying under a N_2_ gas. Samples were washed with isopropanol thrice before dissolving in 500 μl of 0.1 mM cellobiose as an internal standard. A standard sugar mixture (0.2 mM each of fucose, rhamnose, arabinose, galactose, glucose, xylose, mannose, galacturonic acid, and glucuronic acid) was employed for determining molar amounts of individual sugars by single point internal standard quantification. All samples along with standards were subjected to high- HPAEC-PAD on a Dionex ICS-500 instrument. Monosaccharide compositions were calculated as averages (+/− standard error) of biological triplicates and are displayed as molar percentages.

### 2.4 Seed set evaluation

For seed set evaluation, mature siliques were harvested from five-week-old plants and were dissected to identify aborted seeds. For fertilization analysis, self-crossed pistils at floral bud stage 14 were used (Smyth et al., 1990). Reciprocal-crosses were performed for floral buds at stage 12 which were emasculated before hand-pollination. Fresh pollen at flower stage 13 was applied to the stigma of emasculated flowers. Pollinated flowers were placed in the growth chamber for 8 to 10 d and subsequently decolorized in 70 % ethanol at 37 °C for 3 d. After clearing, seed-set and silique lengths were examined using a Nikon SMZ1500 stereomicroscope.

### 2.5 Cytochemical staining

For examining whether pollen grains from *Hyp-GALT* mutant plants were viable, Alexander staining was used as previously described (Peterson et al., 2010). In brief, the Alexander stain was prepared by mixing 10 ml of 95 % ethanol, 1 ml of Malachite green (1 % solution in 95 % ethanol), 50 ml of deionized water, 25 ml of glycerol, 5 ml of acid fuchsin (1 % solution in water), 0.5 ml of Orange G (1 % solution in water), and 4 ml of glacial acetic acid in a total volume of 100 ml. Anthers were stained with Alexander stain and heated to just below boiling for 30 s, rinsed with water, and observed with a Nikon Phot-lab2 light microscope.

To observe nuclei and callose, mature pollen grains were stained with DAPI (Regan and Moffatt, 1990). Briefly, freshly prepared DAPI staining solution (by adding 1.5 μl of 1 mg/ml DAPI stock solution to 1 ml of sterile distilled water) was added to pollen grains from floral stage 13 and the images were captured with a Nikon E600 epifluorescence microscope.

For auramine O staining, pollen grains of stage 13 flowers were suspended in 0.1 % auramine O in 50 mM Tris-HCl, pH 7.5 and observed under a Zeiss LSM-510 laser-scanning confocal microscope at Ohio University using the FITC filter set (excitation wavelength of 465 nm and emission wavelength of 540 nm).

### 2.6 Vanillin staining

For vanillin staining, siliques were collected from WT and octuple mutants 3–4 d after pollination and stained with 1 % vanillin (4-hydroxy-3-methoxybenzaldehyde) in 6 N HCl (Liu et al., 2020). The resulting images were captured using a Nikon Photo-lab2 light microscope.

### 2.7 Staining of pollen over the pistils with aniline blue

Arabidopsis flowers (floral stage 14 of self-pollinated pistils or 24hrs after hand pollination of pistils at stage 12) were fixed in 10 % (v/v) solution of acetic acid and ethanol for approximately 12 h, followed by three washes with water (10 min each) and bleaching with 8 M NaOH overnight. The pistils were washed again with water (10 min each), and the material was incubated in a solution of decolorized aniline blue 0.1 % (w/v) in the dark for 3 h. Samples were observed using a Nikon E600 upright epifluorescence microscope.

### 2.8 Scanning electron microscopy, transmission electron microscopy, immunolabelling and toluidine blue staining

Scanning electron microscopy (SEM) and transmission electron microscopy (TEM) analysis, immunolabelling and toluidine blue staining for thick anther sections was performed as described previously in detail by Kaur et al. (2022). Briefly, for SEM observations, pollen grains and anthers of all genotypes were dry-mounted on aluminum stubs using double-adhesive tapes and sputter-coated with a palladium alloy using an Anatech HUMMER 6.2 Sputtering System). Images were acquired using an SEM JEOL JSM-6390, HV/LV Tungsten/LaB6, Jeol USA Inc. (Hitachi High-Technologies, Japan), with an accelerating voltage of 15 kV at the Institute for Corrosion and Multiphase Technology, Ohio University. To perform TEM, ultrathin sections of resin-embedded anthers were prepared using a Leica EM UC6 ultramicrotome (Wetzlar, Germany) with a diamond knife and mounted on copper grids. Specimens were viewed with a Hitachi H-7500 Transmission Electron Microscope equipped with an SIA-L12C digital camera and software at the Molecular and Cellular Imaging Center (MCIC), Ohio State University, OARDC, Wooster, OH.

For immunolabelling, flower buds were fixed in buffer [2 % (v/v) formaldehyde, 2.5 %(v/v) glutaraldehyde, 25 mM Na-P buffer, pH7.5] for 24 h at 4 °C, then dehydrated through an ethanol series. The ethanol was replaced with a 1:1 mix of LR White resin (type medium; Electron Microscopy Sciences) and ethanol, then with pure resin. Sections (1 μm) were cut in a microtome and mounted on MAS-coated glass slides. Sections were treated with a solution (1 % (w/v) bovine serum albumin (BSA) in PBST (5.1 mM Na_2_HPO_4_, 1.6 mM KH_2_PO_4_, 130 mM NaCl, 0.02 % Tween 20) for 1 h at RT for blocking, and subsequently incubated with a 1:10 dilution of primary antibody (JIM13 from Kerafast Inc., Catalogue No. ELD025, Lot No. 201203, LM2 from Plant Probes, Catalogue#PP-003) in the same buffer. PBST buffer washing was then conducted three times. Alexa Fluor 488 Goat anti-rat fluorescein isothiocyanate (FITC)-conjugated secondary antibody (Invitrogen; diluted 1:100 in PBS in 1 % BSA) was used for a 2 h incubation in the dark at RT. After washing with PBST, slides were mounted with aqua-poly/mount (Polysciences). A Nikon Eclipse E600 epifluorescence microscope was used for observations. Fluorescence of Alexa Fluor 488 and background autofluorescence of the samples were captured with FITC bandpass filter (excitation wavelength of 460–500 nm, emission wavelength of 510–560 nm) and a DAPI bandpass filter (excitation wavelength of 330–380 nm, emission wavelength of 435–485 nm), respectively. Both images were captured simultaneously to make an overlapping image with Photoshop software.

### 2.9 *In vitro* pollen tube germination and staining

Pollen from anthers of open flowers were germinated *in vitro* as described previously (Kaur et al., 2021) and pollen tubes were fixed and immunolabelled as described previously (Dumont et al., 2015). Flowers collected from WT and *Hyp-GALT* mutant plants 1–2 weeks after bolting were used for examination of pollen tube phenotypes. In short, individual open flowers were germinated *in vitro* on a liquid germination medium (0.01 % H_3_BO_3_, 1 mM Ca(NO_3_)_2_, 1 mM KCl, 1 mM CaCl_2_, 10 % sucrose, 0.03 % casein enzymatic hydrolysate, 0.01 % myo-inositol, 0.1 mM spermidine, 10 mM GABA, and 500 μM methyl jasmonate, pH 7.5), and pollen tubes were grown at 22 °C and 100 % humidity in the dark for 6 h for both immunolabeling. Pollen tubes were treated with a fixative medium containing 5 % methanol-free formaldehyde in PBS buffer and were incubated overnight at 4 °C. The pollen tubes were treated with primary antibodies diluted at 1:5 or 1:10 with phosphate-buffered saline, PBS (with 3 % milk) for 1 h at room temperature. Then, pollen tubes were rinsed with the buffer and incubated in the dark with goat anti-rat IgG -FITC secondary antibody (diluted 1:50) for 1 h at room temperature. Controls were carried out by incubation of the pollen tubes with the secondary antibody only.

### 2.10 Ruthenium red staining

Ruthenium red staining of seeds was performed as described by Kaur et al. (2021). Seeds of all indicated genotypes were pre-hydrated in water for 1 h with shaking (200 rpm) to remove non-adherent mucilage and stained with 0.01 % ruthenium red and were examined under a Nikon SMZ1500 stereomicroscope coupled with a CCD Infinity 2 camera.

## 3 Results

### 3.1 Generation of *galt23456789* octuple mutants and their severe vegetative defects

**Fig. 1:**
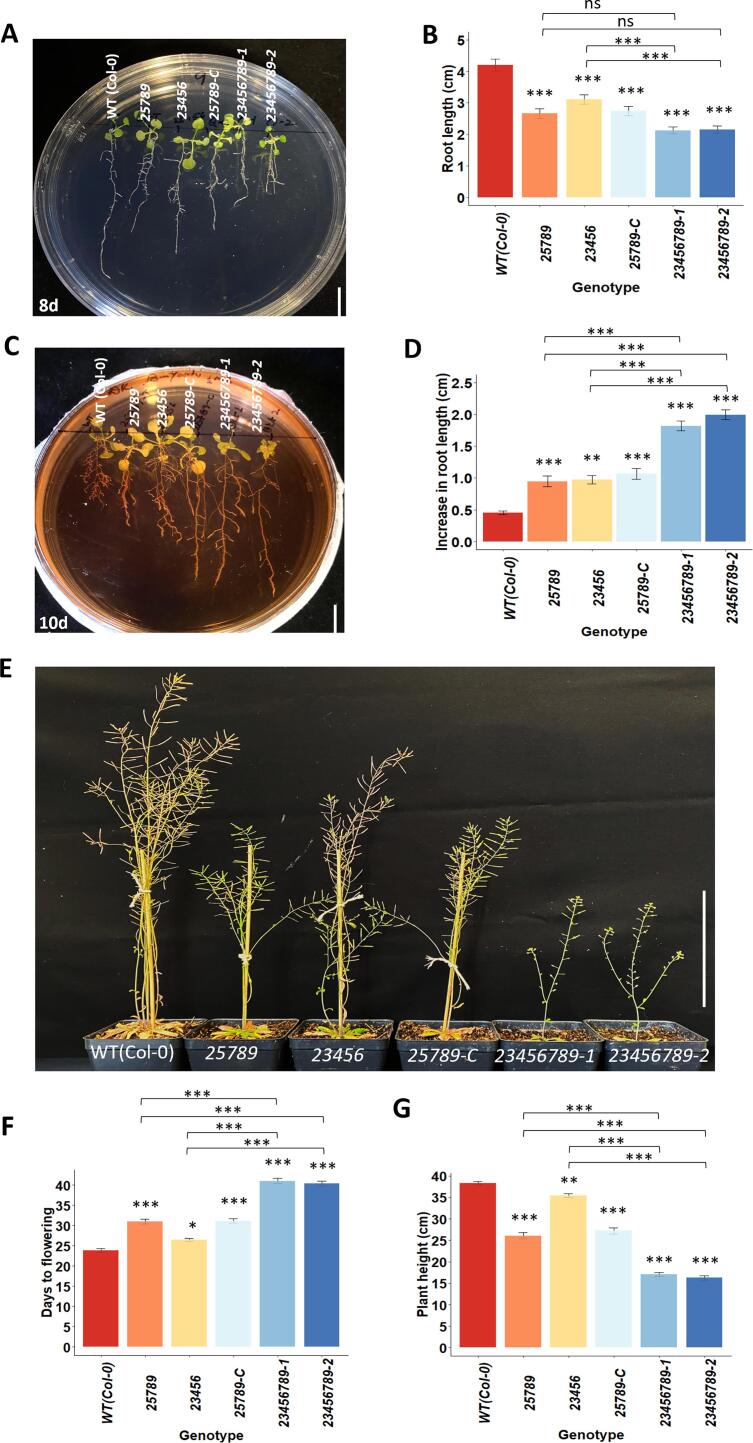
Vegetative growth phenotypes of octuple *Hyp-GALT* mutants in normal MS media, soil and β-Gal Yariv supplemented MS media. (A) Seedlings of the *Hyp-GALT* mutants and WT grown on MS media at 8 d; mean ± SE, *n* = 45 in three independent experiments were quantified in (B). (C) Reduced inhibition of primary root growth of the *Hyp-GALT* mutant seedlings in the presence of β-Gal Yariv reagent. Root lengths were measured at 10 d after transfer to 50 μM β-Gal Yariv supplemented MS and was quantified (mean ± SE) from at least three independent experiments (*n* = 15–20 seedlings in (D). (E) Growth phenotype of the *Hyp-GALT* mutants grown in soil at 50 days after germination (DAG). (F) Days to flowering data in the *Hyp-GALT* mutants grown in soil (mean ± SE, *n* = 30 plants). (G) Plant height at 45 DAG under normal conditions (mean ± SE, *n* = 50–70 plants) from three independent experiments. Asterisks represent the statistical significance between genotypes through one way ANOVA and Tukey’s HSD test (*, P < 0.05; **, P < 0.001; ***, P < 0.00001) within a treatment group in comparison to WT. DAG, days after germination. Scale bar = 1 cm in (A, B), 5 cm in (E).

Previously, our lab characterized *galt2galt5galt7galt8galt9* T-DNA quintuple mutants (*galt25789*) (Kaur et al., 2022, Moreira et al., 2023) and *galt2galt3galt4galt5galt6* CRISPR-Cas9 quintuple mutants (*galt23456*) (Zhang et al., 2021) with respect to their reproductive functions. To further examine the functional roles of all eight known *Hyp-GALT* genes, we utilized a CRISPR/Cas9 gene multiplexing approach to target the three *GALT* genes, namely *GALT3*, *GALT4*, and *GALT6* (with four gRNA targets previously reported by Zhang et al., 2021) in a *galt25789* quintuple T-DNA mutant background (Fig. S1A, S1B, Table S1). We identified two CRISPR/Cas9 edited lines in T3 generation; D35-3–1 (hereafter referred to as *galt23456789*-1) that resulted in pre-mature stop codons for all four targets, and D46-6–2 (hereafter referred to as *galt23456789*-2) was predicted to have pre-mature stop codons for two genes, *GALT3* and *GALT4,* and a three base pair deletion in *GALT6* resulting in the absence of an isoleucine residue in the GALECTIN domain (Fig. S2, S3). Off-target sites predicted by CRISPR-P 2.0 software above the “Off-score” threshold ≥ 0.05 were sequenced in the T3 generation (Fig. S4, Table S2, S3). A *galt25789*-background mutant T0 line containing Cas9 but no editing after transformation was also identified (named as *25789*-C) and used as a negative control. Both octuple mutant lines experienced significantly more retarded seedling growth (Fig. 1A, 1B) and more insensitivity to β-Gal Yariv growth inhibition (Fig. 1C, 1D) compared to the two quintuple mutants, *galt23456* and *galt25789*. The octuple mutants, *galt23456789*-1 and *galt23456789*-2 flowered at an average of 41 d and 40 d compared to 32 d in *galt25789* and 27 d in *galt23456* quintuple mutants, whereas WT flowered at 24 d (Fig. 1E, 1F). Late bolting resulted in reduced plant height (at 45 d; Fig. 1G).

### 3.2 Reduction in β-Gal Yariv precipitated AGPs and molar percentages of galactose and arabinose in *galt23456789* mutants

**Fig. 2:**
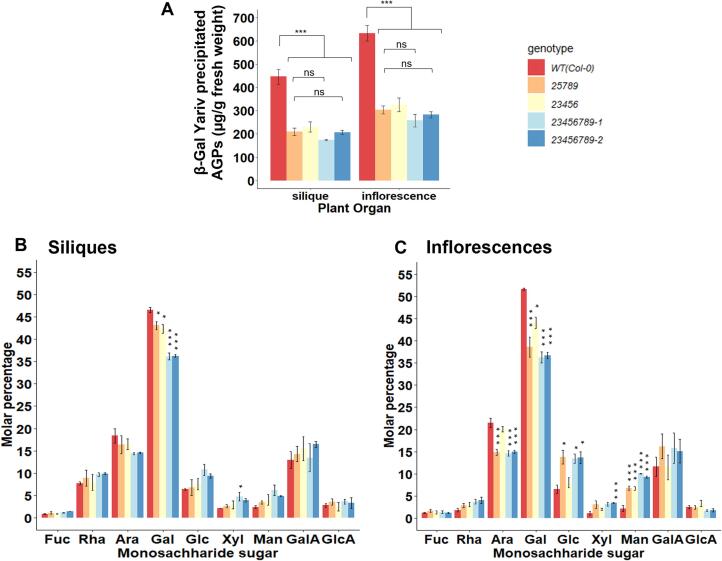
Amount of β-Gal Yariv precipitated AGPs and monosaccharide composition analysis of AGPs isolated from siliques and inflorescences of the *Hyp-GALT* mutants. (A) β-Gal Yariv precipitated AGPs from *23456789-1* and *23456789*-*2* octuple mutants showed comparatively the highest reductions in AGP content from siliques and inflorescences followed by other quintuple mutants. Values represent mean ± SE. Asterisks indicate significant differences compared to the WT according to student’s *t* test (*, P < 0.05, **, P < 0.001, *** P < 0.000001; n = 3). (B and C) Monosaccharide composition analysis of AGPs isolated from siliques and inflorescences of the *Hyp-GALT* mutants. Values represent mean ± SE, *n* = 3. One way ANOVA and post hoc Tukey test was applied to determine significant differences from WT. *P < 0.05, **P < 0.01, ***P < 0.001.

Both *23456789*-*1* and *23456789*-*2* octuple mutant inflorescences displayed 55 % and 59 % reductions in β-Gal-Yariv precipitated AGPs respectively, compared to the *25789* and *23456* quintuple mutants which had 52 % and 49 % reductions relative to the WT (Fig. 2A). Similarly, for silique tissues, *23456789*-*1* and *23456789*-*2* displayed 54 % and 61 % decreases in precipitated AGPs compared to 53 % and 49 % reductions in the *25789* and *23456* quintuple mutants (Fig. 2A). Based on monosaccharide composition analysis, the inflorescence and silique β-Yariv precipitated AGPs were mainly composed of Gal and Ara residues (2.2–2.6:1 M ratio of Gal to Ara). Relative to the WT AGPs, a more pronounced decrease in Gal content of the *23456789*-*1*, *23456789*-*2* octuple mutant (∼23 %, 23 % in siliques; ∼29 %, 30 % in inflorescences) was observed in comparison to the *25789* and *23456* mutants (∼8%, 9 % in siliques; ∼25 %, 15 % in inflorescences) (Fig. 2B and 2C). Similarly, the Ara content was also reduced in *23456789*-*1*, *23456789*-*2, 25789* and *23456* mutants (∼23 %, 22 %, 11 %, 10 % in siliques and ∼32 %, 30 %, 31 %, 6 % in inflorescences, respectively). Given that the *23456789*-*2* mutant line exhibited only one amino acid deletion in the GALT6-3 protein, we used the *23456789*-*1* mutant line (with predicted premature stop codons for all three targeted GALTs) for further functional characterization and hereafter refer to it as the *23456789* mutant.

### 3.3 *galt23456789* mutants have impaired reproductive organ development and dramatically reduced seed set

**Fig. 3:**
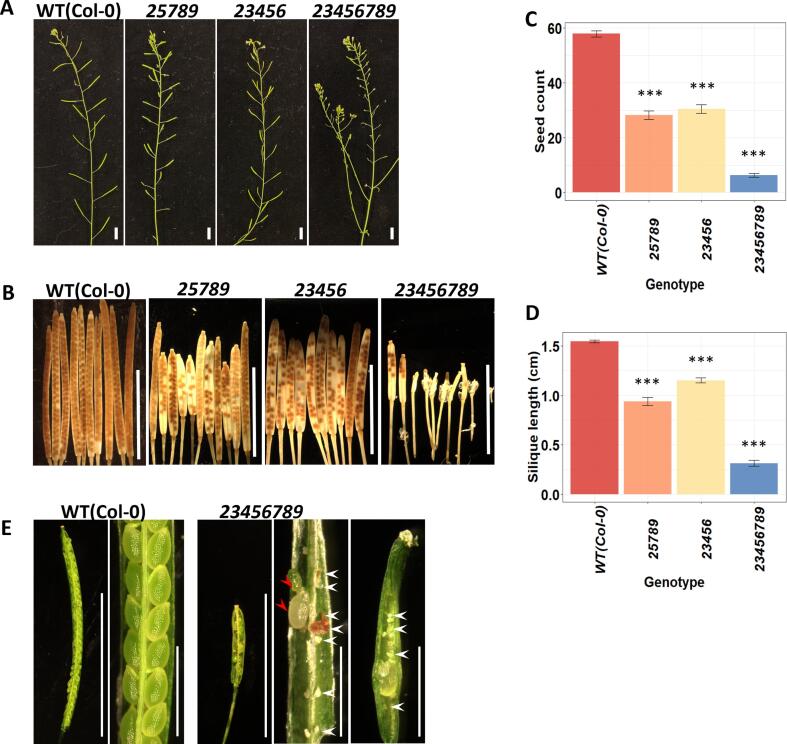
Seed set showed severe defects in the *23456789 Hyp-GALT* mutants. (A) Representative inflorescences of the WT, *25789*, *23456 Hyp-GALT* mutants at 42 DAG and the *23456789* mutant at 50 DAG. (B) *23456789* octuple mutants showed much more severe defects in seed set compared to the low seed sets observed in the *25789* and *23456* quintuple mutant siliques. (C and D) One way ANOVA and post hoc Tukey test were used for statistical analysis, *P* < 0.001, *n* = 50 siliques per genotype, error bar marks mean ± SE. (E) Representative siliques from the WT and *23456789* mutant. White arrowheads indicate aborted seeds that failed to develop; red arrowheads indicate seeds that aborted at a later stage. DAG, days after germination. Scale bars = 1 cm in (A, B, E).

**Fig. 4:**
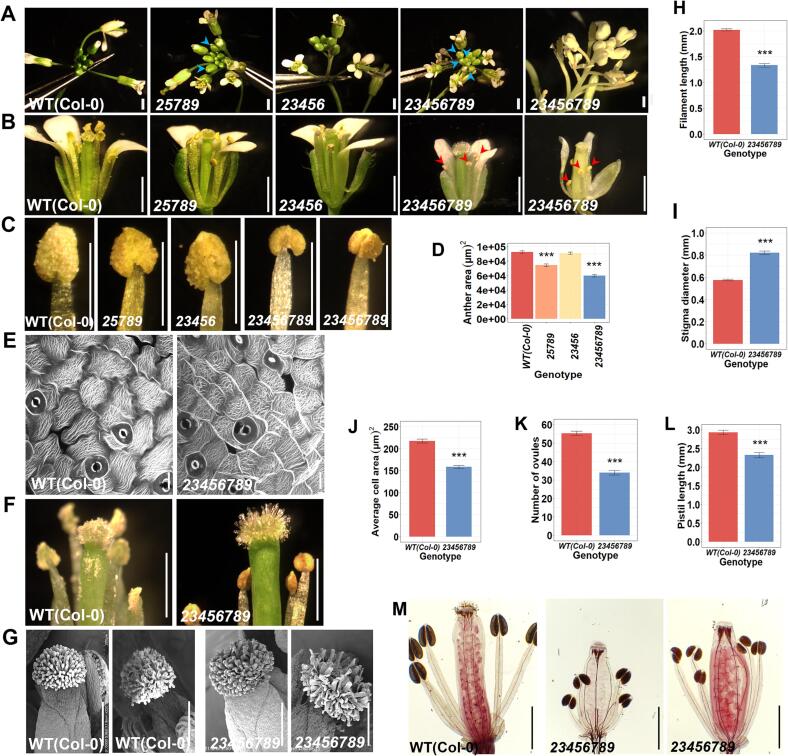
Inflorescence, mature flower androecium and gynoecium morphology of quintuple and octuple *Hyp-GALT* mutants. (A, B and C) Inflorescence, flower and anther morphology at stage 14 for WT, *25789*, *23456* and *23456789*. Abnormal floral organ development shows early opening of floral buds in *25789* and more so in *23456789* mutants (blue arrowheads). Shorter filaments bearing smaller late-dehiscent anthers (red arrowheads) in the *23456789* mutant with enlarged anther view in panel C. (D) Anther area in the *25789* quintuple mutant and the *23456789* octuple mutant was significantly decreased. (mean ± SE, *n* = 30 anthers from 10 flowers of each genotype). One way ANOVA and a post hoc Tukey test was applied to see significant differences from WT, ***p < 0.001. (E) Confocal micrographs of the abaxial side of anthers at flower stage 12 and (J) shows the comparison of average anther cell area in flower stage 12 of WT and *23456789* (mean ± SE, n = 200 cells from 10 anthers of each genotype, ***p < 0.001). (F) Side view (G) and top view of WT and *23456789* stigmas at stage 12 and 14. (H) Filament length is significantly smaller in *23456789* mutant stamens than the WT (mean ± SE, n = 50 filaments from 10 to 12 anthers, ***p < 0.0001). (M) Representative images of aceto-carmine staining of gynoecia from WT and the *23456789* mutant. Sepals, petal and some anthers were removed before fixation and aceto-carmine staining. (I, K and L) Quantification of stigma diameter, pistil length and number of ovules at floral developmental stage 12–13 of WT and the octuple mutant. Scale bars = 500 µm in (A, C, G), 1 mm in (B, F, M), and 10 µm in (E).

We observed morphological alterations in the inflorescences, buds, open flower, androecium, and gynoecium of the *23456789* mutants. The octuple mutant demonstrated more severe reduction in seed set (∼86 %) than the *25789* and *23456* quintuple mutants that showed 57–60 % reductions compared to the WT (Fig. 3A-3C, 3E). Silique length was reduced by ∼64 %, 41 % and 26 % in the *23456789, 25789,* and *23456* mutants, respectively, relative to the WT (Fig. 3B, 3D, Fig. S5). Compared to the WT, the *23456789* octuple mutant had compact inflorescences with a greater number of flowers at the apical ends (Figs. 3A, 4A). All eight Hyp-GALT genes are highly expressed in the floral meristem and flower developmental stages according to Smyth et al. (1990) and Alvarez-Buylla et al. (2010) (Fig. S6). Lack of synchronization in floral organ development accompanied the reduction in flower size of the octuple mutants (Fig. S7). Interestingly, sepals opened prior to complete elongation of petals and stamens during bud stage 9–10 in the octuple mutant (Fig. 4A), while WT sepals opened after bud stage 12 when petals became similar in length to the stamens (Fig. 4B, Fig. S7B, S7C). The octuple mutants were semi-sterile having ∼34 % shorter filaments falling short of the receptive stigma upon flower opening (mean length of *23456789* = 1.33 ± 0.15 mm; WT = 2.02 ± 0.09 mm; Fig. 4B, 4H) and ∼36 % smaller anthers (mean area of *23456789* = 59,588.95 µm2; WT = 92,543.09 µm2; Fig. 4C, 4D, 5A) than the WT at stage 12. The *25789* quintuple mutants also displayed a 21 % smaller anther size (Fig. 4C, 4D), while no significant change in anther size was observed in the *23456* quintuple mutants (Fig. 4C, 4D). To ascertain the underlying cause of the small anther size in the *23456789* mutants, we compared the average cell size of the abaxial side of anthers at the same anther developmental stage before dehiscence. The average anther cell size was significantly smaller in *23456789* anthers (WT = 217.24 ± 38.15 µm^2^; *23456789* = 158.51 ± 26.36 µm2, n = 200, ***p < 0.001; Fig. 4E, 4J).

Given that female gametophytic defects were more pronounced in the quintuple mutants, we investigated female reproductive organ phenotypes. The stigma area showed a 44 % increase in *23456789* octuple mutant flowers (0.82 ± 0.05 mm) compared to the WT (0.57 ± 0.03 mm; Fig. 4F, 4G, 4I). Pistil length was reduced by 20 % in *23456789* octuple mutant flowers (2.32 ± 0.22 mm) compared to the WT (2.93 ± 0.34 mm; Fig. 4L). Furthermore, the number of ovules per silique in stage 12–13 in the shortened pistils of the octuple mutants demonstrated a significant 38 % decrease with an average of 32.6 ± 5.9 ovules relative to 52.7 ± 4.3 ovules in the WT (Fig. 4K, 4M). These results indicate that both male and female gametophytic and/or sporophytic defects contribute to the low yield in both quintuples and octuple Hyp-GALT mutants albeit to different extents.

### 3.4 *galt23456789* octuple mutant has severely disrupted reticulate exine structure, tectum patterns, and pollen viability

**Fig. 5:**
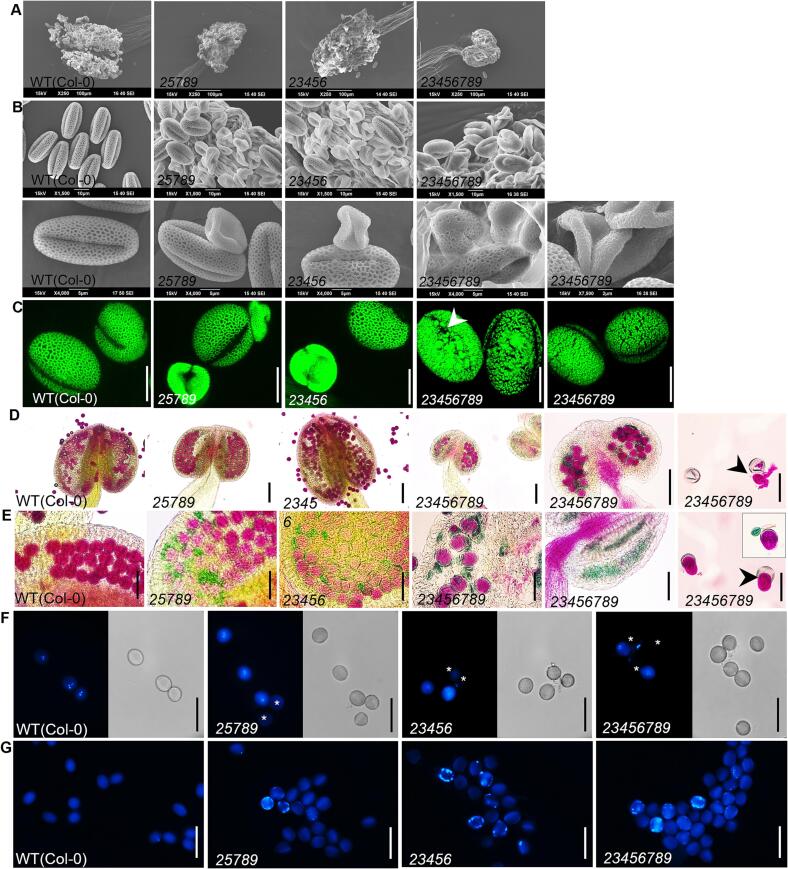
Comparative analysis of anthers and mature pollen grains of *Hyp-GALT* mutants and WT by SEM, auramine-O, Alexander, DAPI, and aniline-blue staining. (A) SEM images of anthers from WT, *25789* and *23456* and *23456789* showed that anther size is greatly reduced in the *25789* and *23456789 GALT* mutants. Anther dehiscence is severely delayed in the *2,345,689* octuple mutant. (B) SEM images of the WT, *25789*, *23456* and *23456789* pollen grains. Aborted pollen grains are visible in the quintuple and octuple mutants (C) With 0.1 % auramine-O stain, WT showed the regular reticulate exine structure details of exine whereas *25789* and *23456* pollen grains displayed some misshaped pollen with abnormal exine patterns. Octuple mutant pollen grains showed a collapsed and defective pollen with a tight reticulate mesh, smaller lacunae, broken exine patterning and flattened apertures (white arrow). (D and E) Alexander stain for pollen viability of *23456789* was greatly reduced compared to WT, *25789* and *23456* mutants. Degenerating pollen grains (black arrowheads) releasing their cellular contents from the exine cell wall of *23456789* mutant pollen grains. Normal pollen appears dark pink (green walls and densely staining cytoplasm) while aborted pollen is marked by empty green walls only (inset in E). (F) DAPI staining of mature pollen grains have two sperm nuclei and one vegetative nucleus in WT, *25789, 23456* and *23456789* but the aborted pollen grains (indicated by white asterisks). (G) Aniline blue staining for callose showed higher intensity in *25789*, *23456* and *23456789*. Scale bars = 100 µm in (A), 10 µm 5 µm and 2 µm as indicated in (B), 10 µm in (C), 200 µm in (D) 50 µm in (E, F, G).

Previously, our work determined that the underlying reasons for the low yield of the *galt25789* mutant were largely contributed by female/embryo developmental defects (Moreira et al., 2023) accompanied by temperate male/microspore developmental defects (Kaur et al., 2022). In contrast, hand-crossing and phenotypic examination of the octuple mutants in this study indicate a major role of the male gametophytic defects causing dramatically low seed set. Anther size, pollen releasing capacity, and pollen viability defects were more acute in the *23456789* octuple mutant than in the *25789* mutants. Additionally, octuple mutant flowers showed delayed dehiscence (Fig. 5A, 5B). In comparison to the abnormal exine patterns of *25789*, more severe distortions in the reticulate exine pattern were observed in the octuple mutant, which had narrower reticulate design with extremely small lacunae and an irregular pollen surface (Fig. 5B, Fig. 5C).

Alexander staining showed a large proportion of non-viable (∼66.7 %; 589/883) pollen in the *23456789* mutants compared to the viable WT pollen (Fig. 5D) while the *25789* and *23456* quintuple mutant showed milder reductions in pollen viability of ∼28.2 % and ∼30 % respectively (Kaur et al., 2022, Zhang et al., 2021). In severe instances, all pollen grains in an anther were non-viable (Fig. 5E) and appeared to lose their cytoplasmic content leaving behind exine remnants (Fig. 5D and E). These observations were confirmed by DAPI staining which revealed mature pollen grains lacking the two sperm nuclei and the vegetative nuclei in most of the pollen grains (Fig. 5F). Aniline blue staining of the *23456789* mutant microspores at stage 12 and the anther endothecium wall (Fig. 5G; Fig. S8) showed intense callose staining in comparison to the WT.

### 3.5 Abnormalities in the sporophytic tapetal layer and intine and exine layer of pollen grains in *galt23456789* octuple mutants

**Fig. 6:**
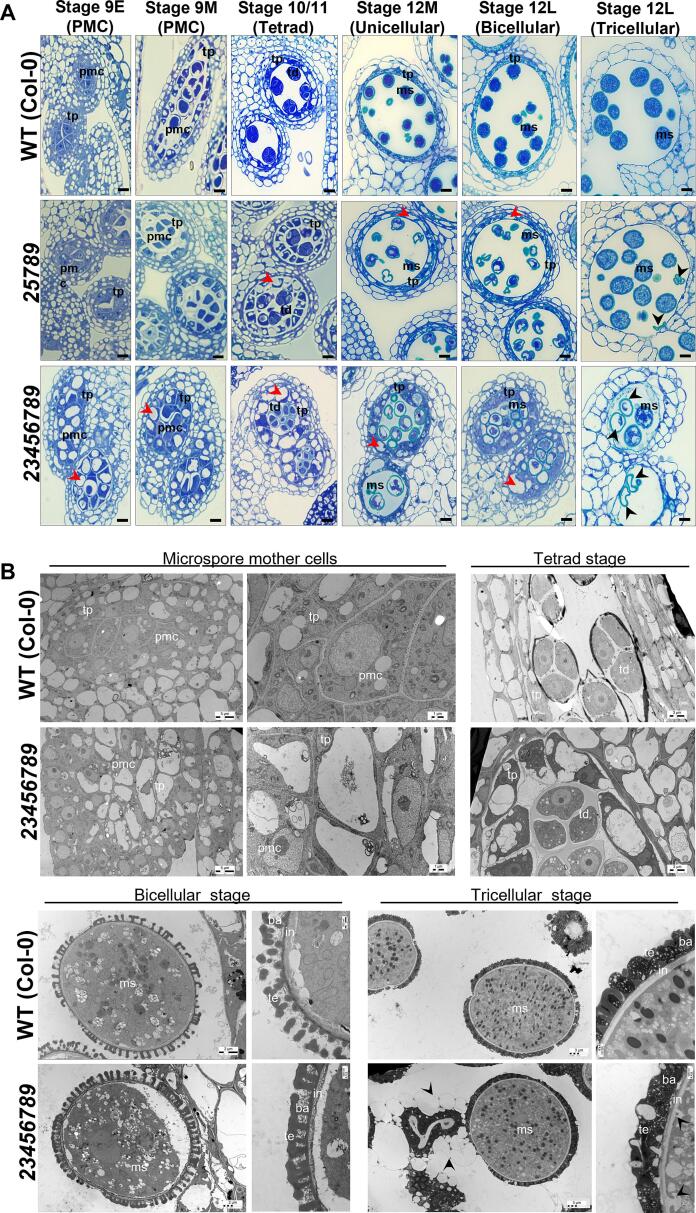
Defects in pollen development of the *Hyp-GALT* mutants. (A) Light micrographs of cross-sections of resin-embedded anthers of WT, *galt25789* and *galt23456789* octuple mutant plants stained with toluidine blue. All the stages show vacuolated and thicker tapetal cell layer as well as vacuolated pollen grains at binucleate stage of the *galt23456789* mutant. Cytoplasmic shrinkage of pollen grains occurs at bicellular stage. Stage 12L shows delated dehiscence and cell wall debris of crushed mutant pollen grains. (B) TEM images from *galt23456789* mutant plants show numerous large vacuoles and starch granules in the pmc stage which are not visible in WT. The large vacuoles in the tapetum layer persist during the tetrad stage. The bicellular stage in mutants shows a thin intine. Membrane blebbing, weaker intine and cellular debris are evident in the tricellular stage of *galt23456789* mutant microspores (indicated by black arrows). The aborted pollen grain collapses and degrades cellular content and nuclei. Scale bar = 10 µm in (A), 5 μm; 3 μm; 2 μm; 500 nm as indicated in (B). pmc-pollen mother cell; td-tetrad; tp-tapetum; ms-microspore; ba, baculum; ex, exine; in, intine; te, tectum; pc-pollen coat.

Next, we examined cytological differences in pollen grain development between the WT, *25789* and *23456789* mutant using toluidine blue staining of anther sections. The octuple mutant anthers showed the presence of large and numerous vacuoles in differentiating pollen mother cells, in the sporophytic tapetum layer throughout development, and during the maturation process of the microspores (Fig. 6A) which was not observed in *25789* mutant. The tapetum layer of anthers in both mutants also appeared swollen, more so in octuple mutant. In addition, the tapetal layer degenerated later in the octuple mutant compared to WT. While both quintuple and octuple mutant developing microspores presented abnormal cytoplasmic vacuoles and cytoplasmic shrinkage, the defects intensified in the octuple mutants, thereby, suggesting the additive effect of Hyp-GALT knockouts. At the tricellular stage (12L), the tapetal layer is not present in WT anther locules, while the *23456789* mutant locules showed mature viable and collapsed pollen grains (12L) muddled up, possibly in the debris released by the collapsed pollen grains and/or the tapetum layer remains (Fig. 6A).

Transmission electron microscope (TEM) imaging provided further insight to the differentiation and development of microspores (Fig. 6B). The tapetal cells surrounding the pollen mother cells (pmc) in the mutant displayed large lytic vacuoles, reflecting abnormal metabolic activity when compared to the WT. In line with previous observations, TEM imaging showed that tapetum degeneration is delayed and/or aborted pollen grain cell contents are released into the anther locule in the *23456789* mutant such that the complete degradation is achieved by flower stage 12L-13 compared to 12M stage in WT. While the intine layer was under-developed at the bicellular stage of *23456789* mutants, the exine structure presented a tight reticulate meshwork, with more baculae and an overdeveloped tectum.

Overall, octuple mutant anthers showed distinct defects like large aggregates in lytic vacuoles, abnormal microspore intine development, and a swollen tapetum layer during the transition from microspore mother cells to mature microspores (Fig. 6B).

### 3.6 Anther dehiscence delayed and pollen release impeded in *galt23456789* octuple mutants

**Fig. 7:**
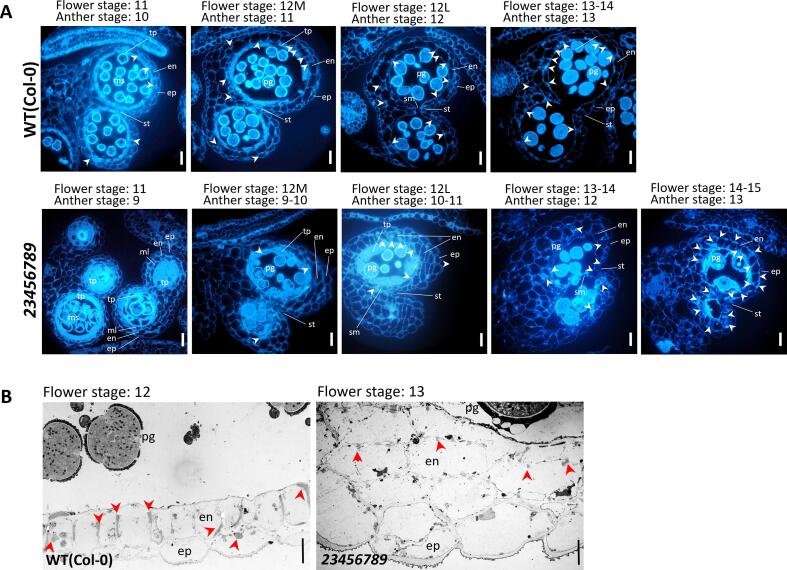
Late dehiscent anthers have delayed tapetum degeneration and delayed lignification in irregularly laid endothecium layers of the *23456789* octuple mutant flowers. (A) Auto fluorescence images of WT and *23456789* anther sections displayed fully dehiscent anthers of WT at flower stage 13–14 and delayed dehiscence in *23456789* anthers by flower stage 15–16. Flower stage 11: endothecium lignification starts in WT anthers (arrowheads) but are absent in the endothecium of the *23456789* mutant. Flower stage 12: lignification is completed (arrowheads) and initial septum lysis is observed in WT anthers. A diffused fluorescence signal for lignification appears in the *23456789-*anther endothecium by flower stage 12M-12L. Also, tapetum degeneration is delayed in *23456789* that also has thicker endothecium layers. Flower stage 13–14: Stomium lysis of WT anthers takes place to release mature pollen whereas septum lysis starts in the *23456789* mutant followed by late stomium lysis in mutant flower stage 14–15. (B) TEM images showing delayed lignification (red arrowheads) in the endothecium layer surrounding the anther locule at flower stage 13 of *23456789* compared to the complete lignification of WT at flower stage 12 M. Scale bars = 20 μm (A), 5 μm (B). en, endothecium; ep, epithelium; ml, middle layer; ms, microspore; pg, pollen grains; sm, septum; st, stomium; tp, tapetum.

To gain insight into the pollen releasing capacity of the octuple mutants where anthers dehisce late and release pollen in the form of clumps, we examined the secondary thickening in the endothecium of anther sections. In flower stage 13–14, WT anthers completed dehiscence and released pollen for pollination (Fig. 7A), whereas *23456789* anthers only initiated dehiscence at that flower stage and were able to open by flower stage 15–16. While the deposition of lignified thickenings in the endothecium surrounding the anther locule appeared by flower stage 11 in WT anthers and showed heavy lignification patterns in stage 12L and stage 13, lignification first appeared in *23456789* endothecia by stages of 12M–12L and later intensified in stages 13–15. Consequently, initial septum lysis in the octuple mutant occurs at stage 13–14 followed by stomium lysis in stage 15–16 in contrast to the same events occurring at stage 12L and stage 13 in WT anthers. Interestingly, anther locules of the octuple mutant were protected by a thicker and irregular endothecium at flower stage 12–13, (Fig. 7B) in contrast to the single layered endothecium in WT; thus, providing another potential reason for delayed dehiscence.

### 3.7 Altered distribution of AGP epitopes in developing anthers

**Fig. 8:**
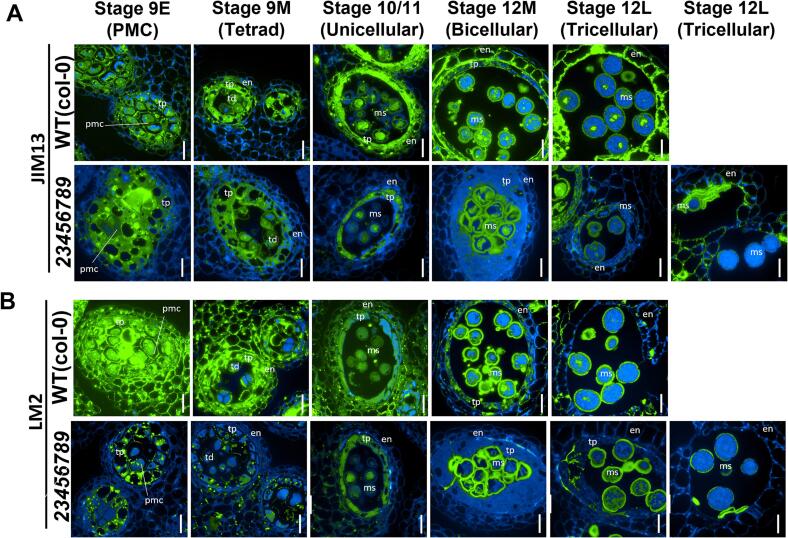
Distribution of JIM13 and LM2-epitope labeling of AGPs in microsporocytes and microspores of *23456789* octuple mutants and WT. Cross sections of resin-embedded anthers of WT and *23456789* from stages 8 to 12L were labelled in green with JIM13 (A) and LM2 (B) primary antibodies followed by Alexa Fluor 488-labeled secondary antibody and the autofluorescence of the cross section is blue. Scale bars = 20 µm. en-endothecium; ep-epithelium; ms-microspore; pmc-pollen mother cell; td-tetrad; tp-tapetum.

Two AGP glycan-specific monoclonal antibodies (mAbs), J1M13 and LM2, were used to examine the amount and distribution of AGPs during Arabidopsis anther development from flower stage 8 to stage 12 M (Pennell et al., 1991, Smallwood et al., 1996, Yan et al., 2015). JIM13, which recognizes AGPs with β-Glc*p*A-(1 → 3)-α-Gal*p*A-(1 → 2)-L-Rha epitopes, produced a distinct labeling pattern for the microspore mother cells and their cell walls, middle layer, tapetal cells, endothecium layers around the anther locules, and walls of the pollen grain in the WT (Fig. 8A). In the anther sections of the *23456789* mutant, JIM13 labelling produced comparatively weaker signals in microspore mother cells and their cell walls and in the tapetal layer. A strong reduction in JIM13 labeling was observed in the endothecium layer of the *23456789* mutants compared to WT. Surprisingly, the aborted pollen grains were strongly labelled in comparison to viable pollen grains. Note that viable pollen grain walls displayed similar labeling in the WT and mutant. LM2, which recognizes AGPs containing β-D-Glc*p*A (Smallwood et al., 1996), labelled the differentiating microspore mother cells and their cell walls as well as pollen grain walls more intensely than the tapetal and endothecium cells in the WT (Fig. 8B).

### 3.8 *galt23456789* octuple mutants had less pollen available for fertilization

**Fig. 9:**
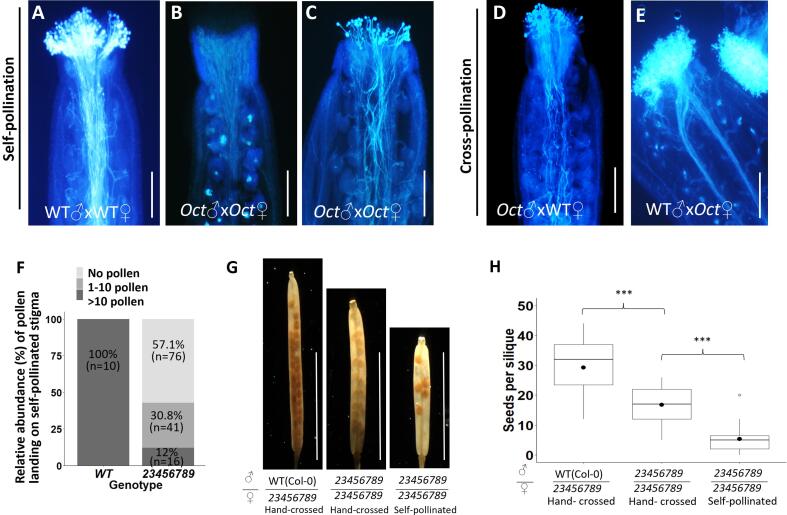
Aniline blue staining of self-pollinated and hand cross-pollinated *23456789* pistils showing the few pollen grains and pollen tubes in *23456789* mutant stigmatic surfaces compared to the normal WT scenario and seed set quantification (A) self-pollinated WT pistil and *23456789* pistils: WT have numerous pollen grains with pollen tubes growing through the transmitting tissue whereas the *23456789* mutant have either no pollen (B) or only a few pollen on the stigmatic surface of self-pollinated pistils (C). (F) Quantification of relative abundance of pollen landing on self-pollinated WT and mutant pistils. Values expressed as percentage (n = 133). (D and E) Whole view of representative aniline blue-stained cross-pollinated WT pistil (with *23456789* pollen) and *23456789* pistils resulting from the cross-pollination (with WT pollen). WT pistils had only a few pollen grains while octuple mutant pistils were extensively covered with WT pollen grains. (G) Representative cleared siliques obtained from hand- crossing and self-pollinated *23456789* octuple mutant pistils. (H) Quantification of the seeds per silique and silique length for cross-pollinated siliques (n ≥ 16 siliques for each cross). Bars represent the median, dots represent the means and asterisks indicate significant differences among crosses between male and female WT plants according to student’s *t*-test ***, P < 0.00001). Scale = 0.5 cm in (G).

Aniline blue staining was performed to analyze the number of pollen grains at the stigma surface area in octuple mutants compared to the WT. Aniline blue staining of self-pollinated *23456789* mutants displayed acute stamen structural defects; 57 % (n = 76/133) of self-pollinated mutant pistils were devoid of any pollen on the stigmatic surface while 31 % had only a few pollen-grains (<10) sticking to the stigmatic area. Strikingly, only 12 % of the octuple mutant pistils had > 10 pollen grains sticking to the stigma (Fig. 9B, 9C, 9F). In contrast, self-pollinated WT plants had a large number of pollen grains attached to the stigmatic area (Fig. 9A). In fact, hand-crossing of *23456789* octuple mutant pistils with *23456789* pollen and WT pollen significantly increased the seed set by three times and five times respectively, compared to self-pollinated *23456789* octuple mutant pistils (Fig. 9D, 9E, 9G, 9H), thereby confirming the scarcity of viable pollen grains for fertilization.

**Fig. 10:**
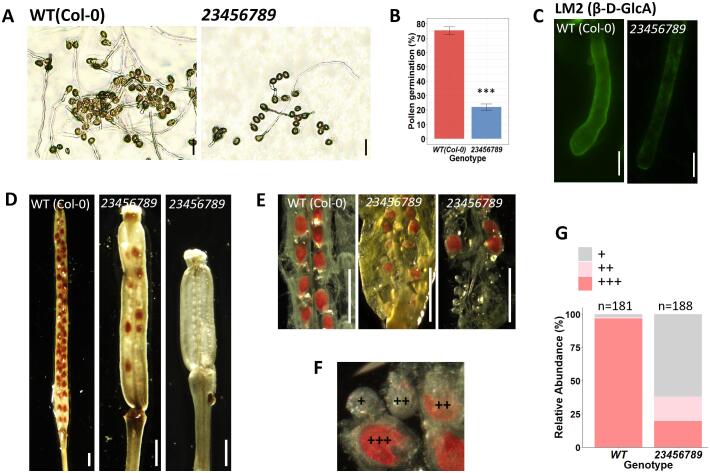
*In vitro* pollen germination, pollen tube immunolabelling and vanillin staining of the *23456789* octuple mutants. (A) Representative images of the *in vitro* pollen assay showing pollen tubes from WT and the octuple mutant in pollen germination medium after 3 h. (B) Quantification of pollen germination. Values expressed as percentages (mean ± SE), asterisks indicate statistically significant, P < 0.005. More than 300 pollen grains for each genotype were measured in three independent experiments. (C) Immunofluorescence labeling of cell wall epitopes probed with anti-AGP (β-D-GlcA) mAb LM2, the signal was weaker in octuple mutant pollen tube tip and shaft. (D, E) Vanillin-stained WT and *23456789* octuple mutant ovules at 3–4 DAP. Scoring for vanillin staining in WT and *23456789* mutant ovules was done with the key in (F) and was plotted in (G). Numbers on the top of the graph (G), indicate total number of ovules counted for both genotypes. Scale bars = 100 µm in (A), 5 µm in (C), 1 mm in (D, E).

Additionally, *in vitro* pollen germination of *23456789* octuple mutant pollen displayed a dramatic decrease in pollen germination (71 %) compared to WT (Fig. 10A, 10B). In mutant pollen tubes, LM2 labeling was greatly reduced compared to WT (Fig. 10C). Vanillin staining is a method used to distinguish fertilization-/ developmental-defective (male and female) gametophytic mutants (Adhikari et al., 2020). Here, the vanillin staining demonstrated 17 % of ovules exhibited fertilization failure, 62 % of ovules remained unfertilized while only 26 % of the ovules were successfully fertilized in the *23456789* mutants (Fig. 10D, 10E, 10F, 10G).

## 4 Discussion

### 4.1 Octuple *Hyp-GALT* mutants display stunted growth and reduced amounts of AGPs

To date, eight *Hyp-GALT* genes encoding enzymes that add the first galactose residues to AGPs have been characterized. Besides the conserved GALT domain (Pf01762) in all *Hyp-GALTs*, the GALT2-6 family members in clade B (subclades V and VI) contain a GALECTIN domain, which is absent in the GALT7-9 family members in clade A (subclade III) of the GT31 phylogenetic tree for Arabidopsis (Qu et al., 2008). Reports characterizing *23456* and *25789* quintuple mutants revealed the overlapping and redundant activities of *Hyp-GALTs* with respect to AGP function for both vegetative and reproductive growth of Arabidopsis (Kaur et al., 2021, Zhang et al., 2021). To understand more about the partially redundant roles of all eight *Hyp-GALTs*, we generated octuple mutants using a CRISPR-Cas9 gene editing approach coupled with T-DNA insertion mutants. Off-target gene mutations were predicted by CRISPR-P 2.0 software. All the off-targets above the “Off-score” threshold ≥0.05 were amplified and sequenced for the resulting *23456789*-*1* and *23456789*-*2* octuple mutants. Although these octuple mutants showed dramatically stunted seedling and plant growth, delayed bolting, and severely reduced seed yield, which is in agreement with previous reports for the pleiotropic effects on vegetative growth in our quintuple mutants (Kaur et al., 2021, Zhang et al., 2021), these knockouts for eight genes were not lethal.

AGPs are implicated to function in various aspects of plant growth and development including cell proliferation and elongation, pattern formation, programmed cell death, cell–cell communication and hormone signaling (Willats and Knox, 1996, Majewska-Sawka and Nothnagel, 2000). AGPs are also associated with the cytoskeleton as indicated by LeAGP1-GFP expression at Hechtian strands in tobacco BY2 cells (Sardar et al., 2006), but other molecular interactors of AGPs defining their cell wall structural and biological properties remain largely unknown. AGPs are also hypothesized to act as cell wall plasticizers, possibly through microfibril separation decreasing cell-wall rigidity or by increasing the porosity of the pectin gel (Rumyantseva, 2005, Lamport et al., 2006). One notable study on *AtAGP57C* provides an example supporting the covalent interaction of an AGP with pectins and hemicelluloses (Tan et al., 2013), while AGP31 presents another example of its PAC domain interacting with rhamnogalacturonan I galactans (Hijazi et al., 2014). Several AGPs (e.g., *FLA10, FLA11*) and COBRA-like proteins (*BRITTLE CULM1*) are highly enriched in secondary cell walls and are thought to modify the physical and chemical properties of the cell walls (MacMillan et al., 2010, Liu et al., 2013). Thus, a reduction in overall AGPs, as seen here in the octuple mutants, likely affects cell wall assembly and wall properties that perturb the mechanics for plant growth.

Comparatively a little more reduction in β-Gal Yariv precipitable AGPs in the octuple mutants compared to the *25789* and *23456* mutants in silique and inflorescence tissues, support the idea Hyp galactosylation is increasingly reduced as more and more *GALT* genes are mutated. However, this reduction was not as pronounced as expected, and could be attributed to severely under-glycosylated AGPs that are still precipitated. Nonetheless, the composition of silique and inflorescence AGPs in the octuple mutants are almost similar to WT AG chains based on monosaccharide composition analysis and our previous biochemical analysis of Hyp-AG chain size (Moreira et al., 2023). Thus, the picture that emerges is that AGPs from the *galt* mutants have normal AG chains, but fewer of these chains attached to AGP protein backbones. Molar percentages of Gal and Ara in the octuple mutant AGPs did show reductions which could reflect reduced activities of other AGP GTs (e.g., β-1,3 galactosyltransferases, β-1,6-galactosyltransferases, and arabinosyl-transferases) potentially interacting with the *Hyp-GALT*s. Thus, future research focusing on AGP GT complexes for type-II AG biosynthesis warrants consideration.

### 4.2 AGP glycosylation is critical for normal reproductive development and functional male reproductive organs

Previously, we observed a reduced fertility phenotype in our higher-order *Hyp-GALT* quintuple mutants (*23456* and *25789*); both male and female gametophytic defects contributed to this phenotype (Zhang et al., 2021, Kaur et al., 2022, Moreira et al., 2023). Here, simultaneous knockout of eight *Hyp-GALT* genes produced large male gametophytic developmental defects along with other abnormal reproductive growth phenotypes, including anisotropic floral organ development and compact inflorescences with smaller flowers. Conspicuous male sporophytic organ defects such as shorter filaments bearing smaller anthers (with reduced average cell sizes) revealed the critical function of Hyp-glycans in the development of male reproductive organs. Several AGPs function in plant reproduction for male gametophyte development, cell division and proliferation, cell differentiation, pattern formation and hormone signaling (Acosta-García and Vielle-Calzada, 2004, Rumyantseva, 2005, Seifert and Roberts, 2007, Levitin et al., 2008, Costa et al., 2019, Leszczuk et al., 2019). For instance, a mutant for a lysine-rich classical AGP (*AGP19)* displayed delayed growth, small inflorescences and rosettes, and low seed set with several abnormalities in cell size, number, shape and packing (Yang et al., 2007). In this study, low amounts of AGPs as detected by β-Gal Yariv precipitation in the octuple mutant inflorescences together with the weak immunolabelling patterns with two AGP-specific mAbs, LM2 and JIM13, indicates a low abundance of AGPs in the sporophytic tissues (specifically the endothecium) of the octuple mutants. Hence, we propose that the smaller cell sizes found in the octuple mutant anthers likely result from changes in cell wall composition and structure due to lack of properly glycosylated AGPs.

Generally, anther locules are surrounded by single cell layered endothecial and epidermal layers and dehiscence occurs by anther wall breakage at the stomium located between the two locules, releasing the pollen grains for pollination and fertilization (Sanders et al., 1999). In the *23456789* octuple mutants, however, a delayed anther dehiscence occurs possibly due to unevenly shaped cells in the two layered endothecial wall layer (likely the remains of the middle layer) and delayed lignification of the endothecium layer which created relatively low-tension during anther wall dehydration. The pollen grains produced by the octuple mutants, albeit with low viability and germination rates, are still able to fertilize octuple mutant pistils. These data along with the mutant pistils largely lacking pollen on self-fertilized stigma is indicative of major sporophytic tissue defects (filament, anther and its endothecium) leading to low seed set in the octuple mutants. Recently, FLA11 and FLA12 have been shown to be involved in regulating the mechanical properties of secondary cell walls in stems, such that type-II AGs are critical for FLA11 function (Ma et al., 2023). Clearly, based on the octuple mutant data, AGP glycans play a crucial role for synchronization of male reproductive organ development by controlling cell wall properties.

Consistent with earlier results of abnormal exine and intine in *25789* quintuple mutant pollen grains (Kaur et al., 2021), we observed even more pronounced defects in the intine and exine patterns in the octuple mutant pollen grains which may render them non-viable. Furthermore, delayed tapetum degeneration and numerous lytic vacuoles in the pollen grains were displayed by the *23456789* mutant compared to WT. Previously, we showed that AGPs are under-glycosylated in inflorescences and that pollen-specific AGPs (AGP1, AGP4, AGP6, AGP25, AGP26, AGP27) are overexpressed in *25789* quintuple mutants (Kaur et al., 2022). However, previously it has been suggested that in the case of excess production of secreted AGPs or insufficiently *O*-glycosylated protein regions only a portion of these AGPs are released from the cell and the rest are shuttled towards vacuolar degradation (Rumyantseva, 2005, Coimbra et al., 2009, Seifert, 2020). Thus, we suggest that the presence of excessive lytic vacuoles observed here presumably marks the internalization of under-glycosylated AGPs.

Other GTs like the anther-specific β-(1,3)-galactosyltransferase (KNS4/UPEX1) responsible for AGP and/or pectin biosynthesis and the glucuronosyltransferases (GLCAT14A/B/C) acting on type-II AGs demonstrated deformed exine structures (Suzuki et al., 2017, Ajayi et al., 2021). While the *kns4*/*upex1* mutant had severe abnormalities in the primexine matrix and exine formation (with smaller lacunae), the *glcat14a/b/c* triple mutants displayed ∼45 % misshaped pollen grains with wider lacuna, sparse baculae and an abnormal intine layer. Compared to these pollen wall abnormalities, we also observed defects in the octuple mutant which were different to some extent such that the tectum was overdeveloped with plentiful baculae (Fig. 6B).

Given that AGPs like AGP6, AGP11, AGP23, and AGP40 constitute an integral part of the intine layer, they have been implicated in pollen ontogenesis and pollen function (Jia et al., 2015). While RNAi mutants of AGP6 and AGP11 demonstrated collapsed pollen grains with condensed cytoplasm, membrane blebbing and the presence of small lytic vacuoles (Coimbra et al., 2009, Coimbra et al., 2010), the RNAi transgenic plants of FLA3 displayed 50 % shrunken non-viable pollen grains with defective pollen intine (Li et al., 2010). More recently, FLA14-overexpressing Arabidopsis plants showed about 39 % aborted pollen grains leading to low seed set (Miao et al., 2021). Taken together, the results of our work are consistent with these previous studies and provides new evidence that the Hyp-*O*-glycans of AGPs are essential for male gametophytic development, and more precisely for the development of the intine and exine layers of pollen grains. Although the exact signaling mechanisms connecting the sporophytic tissues with gametophytic development and fertilization remain elusive, it is likely that Hyp-*O*-glycans of AGPs that are also highly expressed in sporophytic and gametophytic organs (Jia et al., 2015, Pereira et al., 2016) serve to mediate such signaling processes.

To summarize, the *25789* quintuple Hyp-GALT mutant previously demonstrated mild male gametophytic defects and stronger female gametophytic defects (Kaur et al., 2022, Moreira et al., 2023), in contrast the octuple mutant in this study showed that male gametophytic defects (mainly due to shorter stamens reducing anther/ pollen grains to the stigma, acute defects in anther dehiscence and ∼66.7 % reductions in pollen viability) might contribute more to dramatically low yields; while the contribution of female reproductive defects needs further investigation.

## 5 Conclusions

The carbohydrate moieties of AGPs have been long hypothesized to be the components underpinning the biological functions of AGPs, during vegetative and reproductive development. Through this study, we provide strong reverse genetic evidence for abnormal vegetative and reproductive growth phenotypes in the octuple mutants, establishing a clear role for AGP glycans for maintaining normal plant root and shoot growth, male gametophytic and sporophytic organ morphogenesis, cell wall integrity, and AGP-dependent signaling for successful fertilization, ensuring overall yield.

Further investigations, however, are needed to decode the specific biological role of each sugar of the complex AG glycomodules. Exactly how many *Hyp-GALT* genes exist to catalyze the addition of first galactose during AGP Hyp-*O*-galactosylation awaits further analysis. And the signaling pathways that work behind the various AGP functions needs to be discovered. Working towards these directions will help to define the precise roles of AGP Hyp-*O-*glycans in the processes of vegetative and sexual reproduction.

## Funding

DK’s work was supported by 10.13039/100008076Ohio University (OU) Student Enhancement Award; an OU College of Arts and Science Graduate Student Research Fund award; and an OU Nanoscale & Quantum Phenomena Institute (NQPI) fellowship to D.K. DM’s research was supported by an FCT PhD grant (SFRH/BD/143557/2019). SC received finantial support from PT national funds (FCT/10.13039/501100006111MCTES, Fundação para a Ciência e Tecnologia and Ministério da Ciência, Tecnologia e Ensino Superior) through the project UIDB/ 50006/2020).

## CRediT authorship contribution statement

**Dasmeet Kaur:** Writing – review & editing, Writing – original draft, Visualization, Validation, Supervision, Software, Resources, Project administration, Methodology, Investigation, Funding acquisition, Formal analysis, Data curation, Conceptualization. **Michael A. Held:** Writing – review & editing, Methodology, Investigation. **Yuan Zhang:** Writing – review & editing, Software, Methodology, Investigation, Data curation. **Diana Moreira:** Writing – review & editing, Methodology, Investigation. **Silvia Coimbra:** Writing – review & editing, Visualization, Validation, Methodology, Investigation. **Allan M. Showalter:** Writing – review & editing, Visualization, Validation, Supervision, Resources, Project administration, Methodology, Investigation, Funding acquisition, Formal analysis, Data curation, Conceptualization.

## Declaration of competing interest

The authors declare that they have no known competing financial interests or personal relationships that could have appeared to influence the work reported in this paper.

## References

[b0005] Acosta-García G., Vielle-Calzada J.P. (2004). A classical arabinogalactan protein is essential for the initiation of female gametogenesis in Arabidopsis. Plant Cell.

[b0010] Adhikari P.B., Liu X., Kasahara R.D. (2020). Fertilization-defective gametophytic mutant screening: A novel approach. Front. Plant Sci..

[b0015] Ajayi O.O., Held M.A., Showalter A.M. (2021). Glucuronidation of type II arabinogalactan polysaccharides function in sexual reproduction of Arabidopsis. Plant J..

[b0020] Alvarez-Buylla E.R., Benítez M., Corvera-Poiré A., Chaos C.Á., de Folter S., Gamboa de Buen A., Garay-Arroyo A., García-Ponce B., Jaimes-Miranda F., Pérez-Ruiz R.V. (2010).

[b0025] Ariizumi T., Steber C.M. (2007). Seed germination of GA-Insensitive *sleepy1* mutants does not require RGL2 protein disappearance in Arabidopsis. Plant Cell.

[b0030] Basu D., Tian L., Wang W., Bobbs S., Herock H., Travers A. (2015). A small multigene hydroxyproline-O-galactosyltransferase family functions in arabinogalactan-protein glycosylation, growth and development in Arabidopsis. BMC Plant Biol..

[b0035] Basu D., Wang W., Ma S., DeBrosse T., Poirier E., Emch K. (2015). Two hydroxyproline galactosyltransferases, GALT5 and GALT2, function in arabinogalactan-protein glycosylation, growth and development in Arabidopsis. PLoS ONE.

[b0040] Bleckmann A., Alter S., Dresselhaus T. (2014). The beginning of a seed: regulatory mechanisms of double fertilization. Front. Plant Sci..

[b0045] Borner G.H., Lilley K.S., Stevens T.J., Dupree P. (2003). Identification of glycosylphosphatidylinositol-anchored proteins in Arabidopsis. A proteomic and genomic analysis. Plant Physiol..

[b0050] Clough S.J., Bent A.F. (1998). Floral dip: a simplified method for Agrobacterium -mediated transformation of Arabidopsis thaliana. Plant J..

[b0055] Coimbra S., Pereira L.G., 2012. Arabinogalactan proteins in *Arabidopsis thaliana* pollen development. Transgenic Plants - Advances and Limitations. Intech Open Limited, London, UK doi: 10.5772/30833.

[b0060] Coimbra S., Costa M., Jones B., Mendes M.A., Pereira L.G. (2009). Pollen grain development is compromised in Arabidopsis *agp6 agp11* null mutants. J. Exp. Bot..

[b0065] Coimbra S., Costa M., Mendes M.A., Pereira A.M., Pinto J., Pereira L.G. (2010). Early germination of Arabidopsis pollen in a double null mutant for the arabinogalactan protein genes *AGP6* and *AGP11*. Sex. Plant Reprod..

[b0070] Costa M., Pereira A.M., Pinto S.C., Silva J., Pereira L.G., Coimbra S. (2019). *In silico* and expression analyses of fasciclin-like arabinogalactan proteins reveal functional conservation during embryo and seed development. Plant Reprod..

[b0075] Dresselhaus T., Coimbra S. (2016). Plant reproduction: AMOR enables males to respond to female signals. Curr. Biol..

[b0080] Dumont M., Cataye C., Lehner A., Maréchal E., Lerouge P., Falconet D. (2015). A simple protocol for the immunolabelling of Arabidopsis pollen tube membranes and cell wall polymers. Bio-Protoc..

[b0085] Gaspar Y.M., Nam J., Schultz C.J., Lee L.Y., Gilson P.R., Gelvin S.B., Bacic A. (2004). Characterization of the arabidopsis lysine-rich arabinogalactan-protein AtAGP17 mutant (rat1) that results in a decreased efficiency of agrobacterium transformation. Plant Physiol..

[b0090] Goldberg R.B., de Paiva G., Yadegari R. (1994). Plant embryogenesis: zygote to seed. Science.

[b0095] Grienenberger E., Quilichini T.D. (2021). The toughest material in the plant kingdom: an update on sporopollenin. Front. Plant Sci..

[b0100] Higashiyama T., Hamamura Y. (2008). Gametophytic pollen tube guidance. Sex. Plant Reprod..

[b0105] Hijazi M., Durand J., Pichereaux C., Pont F., Jamet E., Albenne C. (2012). Characterization of the arabinogalactan protein 31 (AGP31) of Arabidopsis thaliana. New advances on the Hyp-O-glycosylation of the pro-rich domain. J. Biol. Chem..

[b0110] Hijazi M., Roujol D., Nguyen-Kim H., del Rocio Cisneros Castillo L., Saland E., Jamet E., Albenne C. (2014). Arabinogalactan protein 31 (AGP31), a putative network-forming protein in Arabidopsis thaliana cell walls?. Ann. Bot..

[b0115] Hou Y., Guo X., Cyprys P., Zhang Y., Bleckmann A., Cai L., Huang Q., Luo Y., Gu H., Dresselhaus T. (2016). Maternal ENODLs are required for pollen tube reception in Arabidopsis. Curr. Biol..

[b0120] Jia Q.S., Zhu J., Xu X.F., Lou Y., Zhang Z.L., Zhang Z.P., Yang Z.N. (2015). Arabidopsis AT-hook protein TEK positively regulates the expression of Arabinogalactan proteins for nexine formation. Mol. Plant.

[b0125] Jiao J., Mizukami A.G., Sankaranarayanan S., Yamguchi J., Itami K., Higashiyama T. (2017). Structure-activity relation of AMOR sugar molecule that activates pollen-tubes for ovular guidance. Plant Physiol..

[b0130] Johnson K.L., Kibble N.A.J., Bacic A., Schultz C.J. (2011). A fasciclin-like arabinogalactan-protein (FLA) mutant of Arabidopsis thaliana, fla1, shows defects in shoot regeneration. PLoS ONE.

[b0135] Johnson M.A., Lord E., Malhó R. (2006). The Pollen Tube: A Cellular and Molecular Perspective.

[b0140] Kaur D., Held M.A., Smith M.R., Showalter A.M. (2021). Functional characterization of hydroxyproline-*O*-galactosyltransferases for Arabidopsis arabinogalactan-protein synthesis. BMC Plant Biol*.*.

[b0145] Kaur D., Moreira D., Coimbra S., Showalter A.M. (2022). Hydroxyproline-*O*-galactosyltransferases synthesizing type II arabinogalactans are essential for male gametophytic development in Arabidopsis. Front. Plant Sci..

[b0150] Lamport D. (2013). Preparation of arabinogalactan glycoproteins from plant tissue. Bio-Protoc.

[b0155] Lamport D.T.A., Kieliszewski M.J., Showalter A.M. (2006). Salt stress upregulates periplasmic arabinogalactan proteins: using salt stress to analyse AGP function. New Phytol..

[b0160] Leszczuk A., Szczuka E., Zdunek A. (2019). Arabinogalactan proteins: Distribution during the development of male and female gametophytes. Plant Physiol. Biochem..

[b0165] Levitin B., Richter D., Markovich I., Zik M. (2008). Arabinogalactan proteins 6 and 11 are required for stamen and pollen function in Arabidopsis. Plant J..

[b0170] Li J., Yu M., Geng L.L., Zhao J. (2010). The fasciclin-like arabinogalactan protein gene, FLA3, is involved in microspore development of Arabidopsis. Plant J..

[b0175] Liu L., Fan X. (2013). Tapetum: regulation and role in sporopollenin biosynthesis in Arabidopsis. Plant Mol. Biol..

[b0180] Liu L., Shang-Guan K., Zhang B., Liu X., Yan M., Zhang L., Shi Y., Zhang M., Qian Q., Li J. (2013). *Brittle culm1*, a COBRA-like protein, functions in cellulose assembly through binding cellulose microfibrils. PLoS Genet..

[b0185] Liu X., Wu X., Adhikari P.B., Zhu S., Kinoshita Y., Berger F., Higashiyama T., Kasahara R.D. (2020). Establishment of a novel method for the identification of fertilization defective mutants in *Arabidopsis thaliana*. Biochem. Biophys. Res. Commun*.*.

[b0190] Ma Y., Shafee T., Mudiyanselage A.M., Ratcliffe J., MacMillan C.P., Mansfield S.D., Bacic A., Johnson K.L. (2023). Distinct functions of FASCILIN-LIKE ARABINOGALACTAN PROTEINS relate to domain structure. Plant Physiol..

[b0195] MacMillan C.P., Mansfield S.D., Stachurski Z.H., Evans R., Southerton S.G. (2010). Fasciclin-like arabinogalactan proteins: specialization for stem biomechanics and cell wall architecture in Arabidopsis and Eucalyptus. Plant J..

[b0200] Majewska-Sawka A., Nothnagel E.A. (2000). The Multiple roles of arabinogalactan proteins in plant development. Plant Physiol..

[b0205] Miao Y., Cao J., Huang L., Yu Y., Lin S. (2021). FLA14 is required for pollen development and preventing premature pollen germination under high humidity in Arabidopsis. BMC Plant Biol..

[b0210] Mizukami A.G., Inatsugi R., Jiao J., Kotake T., Kuwata K., Ootani K., Okuda S., Sankaranarayanan S., Sato Y., Maruyama D. (2016). The AMOR arabinogalactan sugar chain induces pollen-tube competency to respond to ovular guidance. Curr. Biol..

[b0215] Mizuta Y., Higashiyama T. (2018). Chemical signaling for pollen tube guidance at a glance. J. Cell Sci..

[b0220] Moreira D., Kaur D., Pereira A.M., Held M.A., Showalter A.M., Coimbra S. (2023). Type II arabinogalactans initiated by hydroxyproline-O-galactosyltransferases play important roles in pollen–pistil interactions. Plant J..

[b0225] Nagahara S., Takeuchi H., Higashiyama T. (2021). Polyspermy block in the central cell during double fertilization of Arabidopsis thaliana. Front. Plant Sci..

[b0230] Nguema-Ona E., Coimbra S., Vicré-Gibouin M., Mollet J.-C., Driouich A. (2012). Arabinogalactan-proteins in root and pollen tube cells: distribution and functional aspects. Ann. Bot..

[b0235] Ogawa-Ohnishi M., Matsubayashi Y. (2015). Identification of three potent hydroxyproline O -galactosyltransferases in Arabidopsis. Plant J..

[b0240] Okuda S., Suzuki T., Kanaoka M.M., Mori H., Sasaki N., Higashiyama T. (2013). Acquisition of LURE-binding activity at the pollen tube tip of *Torenia fournieri*. Mol. Plant.

[b0245] Pennell R., Janniche L., Kjellbom P., Scofield G., Peart J., Roberts K. (1991). Developmental regulation of a plasma membrane Arabinogalactan protein epitope in oilseed rape flowers. Plant Cell.

[b0250] Pereira A.M., Lopes A.L., Coimbra S. (2016). Arabinogalactan proteins as interactors along the crosstalk between the pollen tube and the female tissues. Front. Plant Sci..

[b0255] Peterson R., Slovin J.P., Chen C. (2010). A simplified method for differential staining of aborted and non-aborted pollen grains. Int. J. Plant Biol*.*.

[b0260] Qu Y., Egelund J., Gilson P.R., Houghton F., Gleeson P.A., Schultz C.J., Bacic A. (2008). Identification of a novel group of putative Arabidopsis thaliana β-(1,3)-galactosyltransferases. Plant Mol. Biol..

[b0265] Quilichini T.D., Grienenberger E., Douglas C.J. (2015). The biosynthesis, composition and assembly of the outer pollen wall: A tough case to crack. Phytochem..

[b0270] Regan S., Moffatt B. (1990). Cytochemical analysis of pollen development in wild-type Arabidopsis and a male-sterile mutant. Plant Cell.

[b0275] Rumyantseva N.I. (2005). Arabinogalactan proteins: involvement in plant growth and morphogenesis. Biochemistry (Moscow).

[b0280] Sanders P.M., Bui A.Q., Weterings K., McIntire K.N., Hsu Y.C., Lee P.Y., Truong M.T., Beals T.P., Goldberg R.B. (1999). Anther developmental defects in *Arabidopsis thaliana* male-sterile mutants. Sex. Plant Reprod..

[b0285] Sardar H.S., Yang J., Showalter A.M. (2006). Molecular interactions of Arabinogalactan proteins with cortical microtubules and f-actin in bright yellow-2 tobacco cultured cells. Plant Physiol*.*.

[b0290] Seifert G.J. (2020). On the potential function of type II Arabinogalactan *O*-glycosylation in regulating the fate of plant secretory proteins. Front. Plant Sci..

[b0295] Seifert G.J., Roberts K. (2007). The biology of Arabinogalactan proteins. Annu. Rev. Plant Biol..

[b0300] Seifert G.J., Xue H., Acet T. (2014). The Arabidopsis thaliana Fasciclin like arabinogalactan protein 4 gene acts synergistically with abscisic acid signalling to control root growth. Ann. Bot..

[b0305] Showalter A.M. (2001). Arabinogalactan-proteins: structure, expression and function. Cell. Mol. Life Sci..

[b0310] Showalter A.M., Basu D. (2016). Glycosylation of arabinogalactan-proteins essential for development in Arabidopsis. Commun. Integr. Biol..

[b0315] Showalter A.M., Keppler B., Lichtenberg J., Gu D., Welch L.R. (2010). A bioinformatics approach to the identification, classification, and analysis of hydroxyproline-rich glycoproteins. Plant Physiol*.*.

[b0320] Silva J., Ferraz R., Dupree P., Showalter A.M., Coimbra S. (2020). Three decades of advances in arabinogalactan-protein biosynthesis. Front. Plant Sci..

[b0325] Smallwood M., Yates E.A., Willats W.G.T., Martin H., Knox J.P. (1996). Immunochemical comparison of membrane-associated and secreted arabinogalactan-proteins in rice and carrot. Planta.

[b0330] Smyth D.R., Bowman J.L., Meyerowitz E.M. (1990). Early flower development in Arabidopsis. Plant Cell.

[b0335] Suzuki T., Narciso J.O., Zeng W., van de Meene A., Yasutomi M., Takemura S., Lampugnani E.R., Doblin M.S., Bacic A., Ishiguro S. (2017). KNS4/UPEX1: A Type II Arabinogalactan β -(1,3)-galactosyltransferase required for pollen exine development. Plant Physiol..

[b0340] Takeuchi H., Higashiyama T. (2016). Tip-localized receptors control pollen tube growth and LURE sensing in Arabidopsis. Nature.

[b0345] Tan L., Eberhard S., Pattathil S., Warder C., Glushka J., Yuan C., Hao Z., Zhu X., Avci U., Miller J.S. (2013). An Arabidopsis cell wall proteoglycan consists of pectin and arabinoxylan covalently linked to an Arabinogalactan protein. Plant Cell.

[b0350] Wang K., Guo Z.L., Zhou W.T., Zhang C., Zhang Z.Y., Lou Y., Xiong S.X., Yao X., Fan J.J., Zhu J. (2018). The Regulation of sporopollenin biosynthesis genes for rapid pollen wall formation. Plant Physiol..

[b0355] Willats W.G.T., Knox J.P. (1996). A role for arabinogalactan-proteins in plant cell expansion: evidence from studies on the interaction of β-glucosyl Yariv reagent with seedlings of Arabidopsis thaliana. Plant J..

[b0360] Yan Y., Takáč T., Li X., Chen H., Wang Y., Xu E., Xie L., Su Z., Šamaj J., Xu C. (2015). Variable content and distribution of arabinogalactan proteins in banana (Musa spp.) under low temperature stress. Front. Plant Sci..

[b0365] Yang J., Sardar H.S., McGovern K.R., Zhang Y., Showalter A.M. (2007). A lysine-rich arabinogalactan protein in Arabidopsis is essential for plant growth and development, including cell division and expansion. Plant J..

[b0370] Zhang Y., Yang J., Showalter A.M. (2011). AtAGP18, a lysine-rich arabinogalactan protein in Arabidopsis thaliana, functions in plant growth and development as a putative co-receptor for signal transduction. Plant Signal. Behav..

[b0375] Zhang Y., Held M.A., Showalter A.M. (2020). Elucidating the roles of three β-glucuronosyltransferases (GLCATs) acting on arabinogalactan-proteins using a CRISPR-Cas9 multiplexing approach in Arabidopsis. BMC Plant Biol..

[b0380] Zhang Y., Held M.A., Kaur D., Showalter A.M. (2021). CRISPR-Cas9 multiplex genome editing of the hydroxyproline-*O-*galactosyltransferase gene family alters arabinogalactan-protein glycosylation and function in Arabidopsis. BMC Plant Biol..

[b0385] Zhou Q., Zhu J., Cui Y.L., Yang Z.N. (2015). Ultrastructure analysis reveals sporopollenin deposition and nexine formation at early stage of pollen wall development in Arabidopsis. Sci. Bull..

[b0390] Zhou K., 2019. Glycosylphosphatidylinositol-anchored proteins in Arabidopsis and one of their common roles in signaling transduction. Front. Plant Sci. doi: 10.3389/fpls.2019.01022. 10.3389/fpls.2019.01022 PMC6726743 31555307

