# Supplementary material for: Knockout of eight hydroxyproline-O-galactosyltransferases cause multiple vegetative and reproductive growth defects
Source: Cell Surf. 2023 Nov 25;10:100117. doi: 10.1016/j.tcsw.2023.100117 (PMC10698532; doi:10.1016/j.tcsw.2023.100117)
Supplement: Supplementary data 1 [file mmc1.docx]

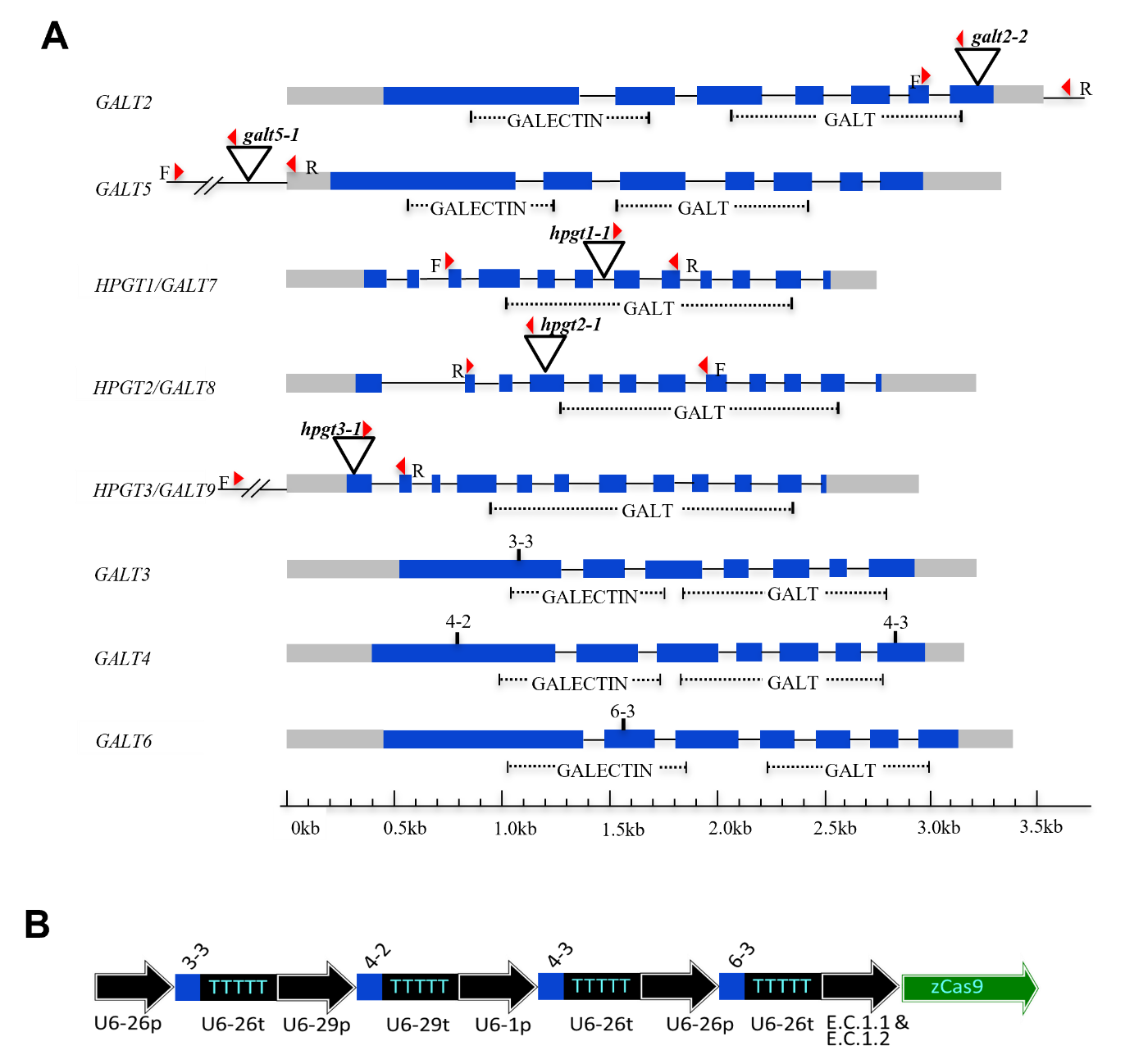
**Supplemental Figures and Tables:**

**Fig. S1. Schematic diagram of eight *GALT* genes showing T-DNA insertions, the guide RNA (gRNA) targeting sites, CRISPR multiplexing construct and efficient gene editing results of target sites *(galt3 galt4 galt6)* in mutant line D35-3-1 and D146-6-2.** (**A**) The quintuple T-DNA insertional mutant for five *GALTs* (*GALT2, GALT5, GALT7, GALT8* and *GALT9*) was generated and confirmed in the previous study by Kaur *et al* (2021), see **Fig. S1**. Four gRNAs were designed to target each one of the three *GALTs* (*GALT3, GALT4,* and *GALT6*). Target sites were labeled as 3-3 for *GALT3*; 4-2 and 4-3 for *GALT4*; and 6-3 for *GALT6*; these gRNAs were cloned in a gene construct shown in (**B**) and were cloned in the pHEE401E vector, which contains a maize codon-optimized *Cas9* gene (*zCas9*) driven by an Arabidopsis egg-cell specific promoter (E.C.1.1) fused with an egg-cell specific enhancer (E.C.1.2). Online software named CRISPR-P 2.0 (<http://crispr.hzau.cn/cgi-bin/CRISPR2/CRISPR>) was used for designing all the gRNAs. Pfam domain predictions: Pf01762 corresponds to the Galactosyltransferase (GALT) domain; Pf00337 corresponds to the Galactose-binding lectin (GALECTIN) domain (<http://www.sanger.ac.uk/Software/Pfam/>). The four targeted gRNA sequences are listed in **Table S2**.


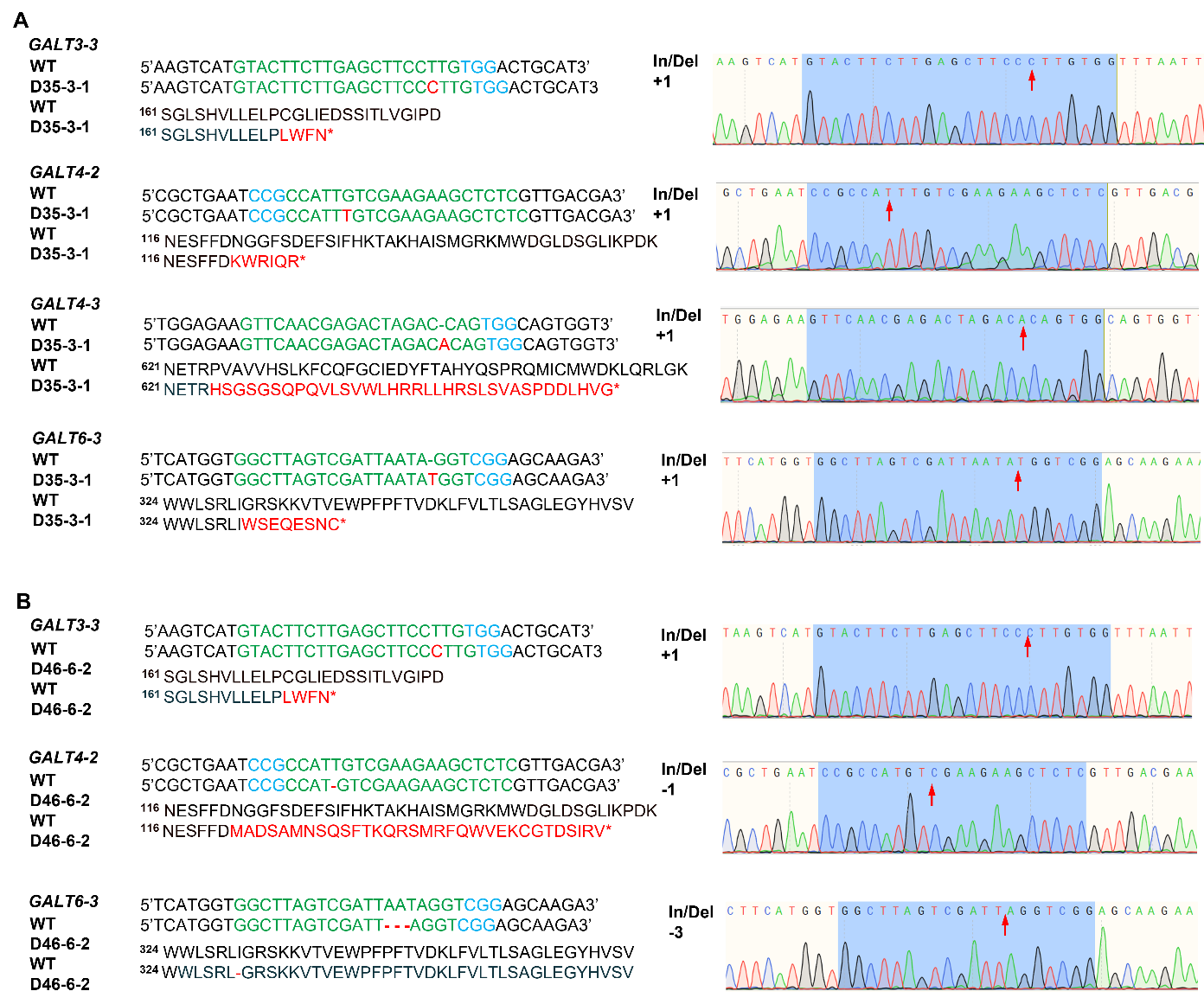


**Fig. S2**: **Efficient gene editing of all given target sites of *galt2 galt3 galt4 galt5 galt6* mutant line *23456789-1* (D35-3-1)** **and *23456789-2* (D-46-6-2).** **A.** Sequencing results for gRNAs in *23456789-1*. (From top to bottom) gRNA (3–3) created a one bp insertion in *GALT3*; gRNA (4-2) created a one bp insertion in *GALT4* and gRNA (4-3) created a one bp insertion in *GALT4*; gRNA (6-3) created a one bp insertion in *GALT6*. All mutations resulted in frame-shifts and pre-mature stop codons. Mutation sites are indicated by red arrows on the sequencing chromatograms on the right. **B.** Sequencing results for gRNAs in *23456789-2*. (From top to bottom) gRNA (3–3) created a one bp insertion in *GALT3*; gRNA targeting 4-2 created a 1 bp insertion in *GALT4*; gRNA (6-3) created a 3 bp deletion in *GALT6*. All mutations resulted in frame-shifts and pre-mature stop codons except 6-3 which resulted in the deletion of one amino acid, isoleucine, in the GALECTIN domain of GALT6. Mutation sites are indicated by red arrows on the sequencing chromatograms on the right.


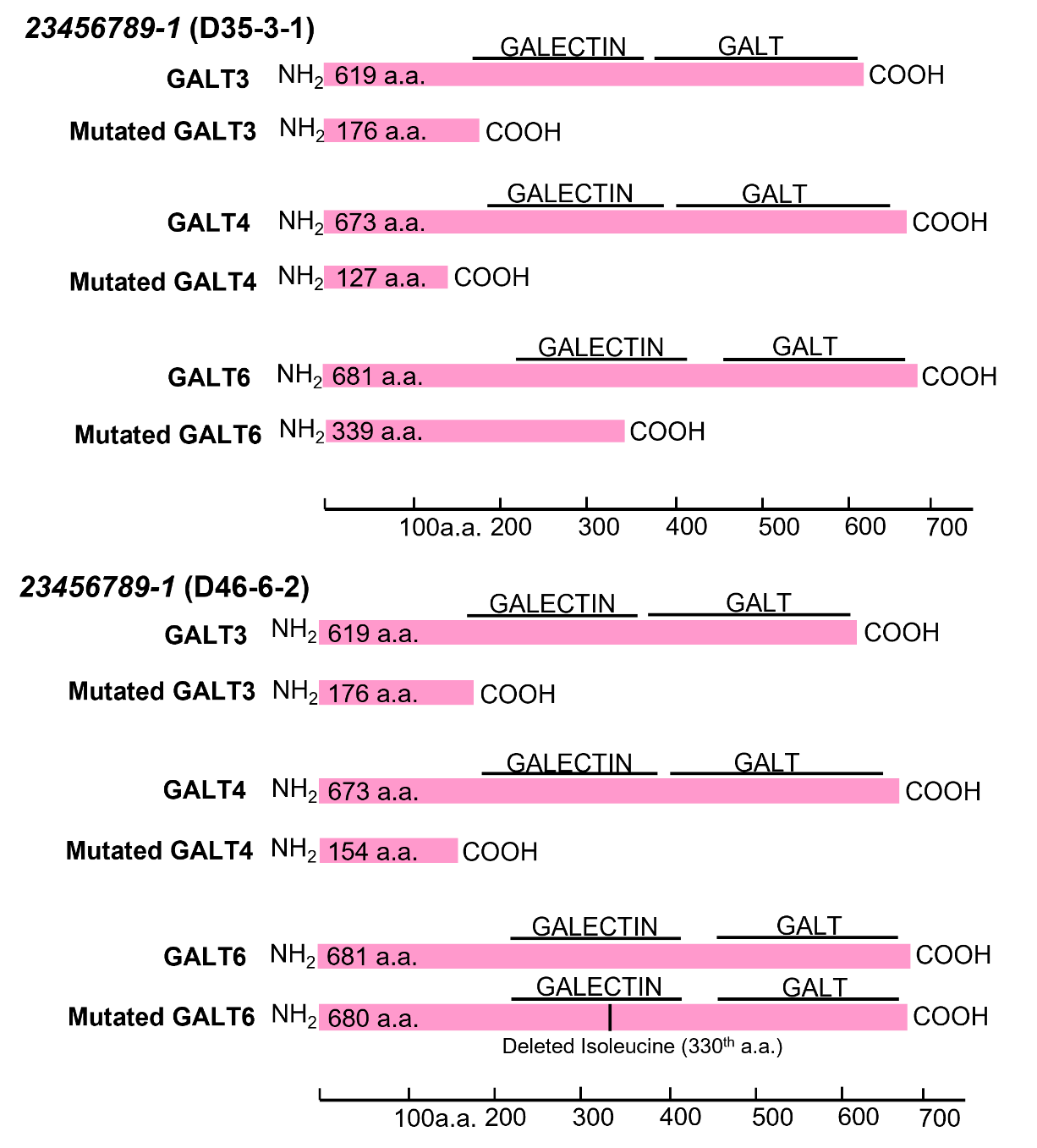


**Fig. S3. Prediction of mutated protein lengths in the CRISPR targeted Hyp-GALTs for *galt23456789-1* and *galt23456789-2* octuple mutants compared to the WT(Col-0).** The normal protein lengths of GALT3, GALT4 and GALT6 are indicated in parenthesis for the WT(Col-0). The *galt23456789-1* is predicted to have mutated GALT3, GALT4 and GALT6 proteins such that they translate into an incomplete GALECTIN domain and no GALT domain in all the mutated GALTs (predicted lengths of mutated GALTs are indicated in parenthesis). The *galt23456789-2* mutant is predicted to have truncated GALT3 and GALT4 proteins such that they both translate into an incomplete GALECTIN domain and no GALT domain, while GALT6 is translated into mutated protein missing one amino acid, an isoleucine at position 330 in the GALECTIN domain.


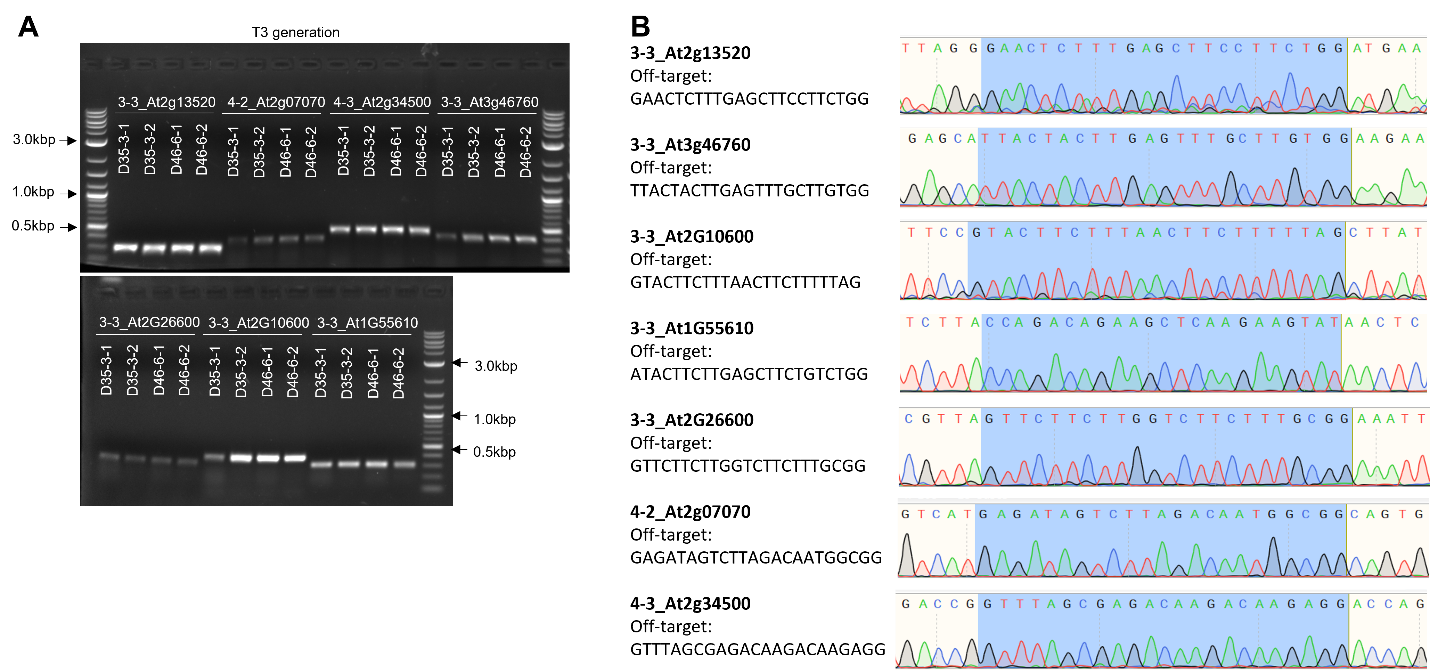
**Fig. S4: Off-targets for *GALT3-3, GLAT4-2, GALT4-3* and *GALT6-3* were checked in T3 generation.** The off targets and sequencing primers are provided in Table S3. **(A)** All the off targets above the “Off-score” threshold ≥ 0.05 were amplified. **(B)** The sequencing results of predicted off-targets for D-35-3-1 are checked and showed no mutation at off-targets.


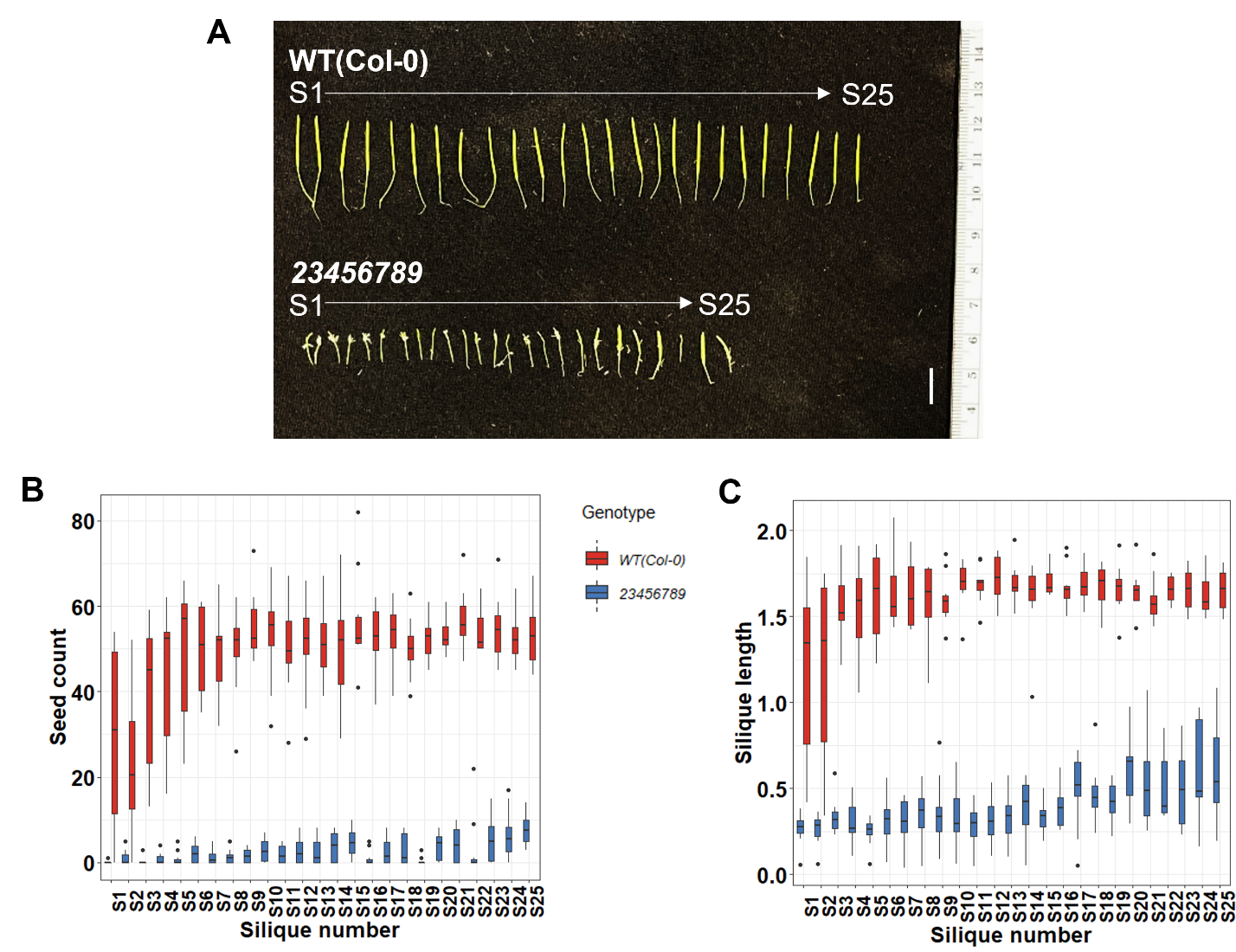


**Fig. S5. Seed set and silique growth phenotype of the *Hyp-GALT* mutants under normal conditions.** (**A**) Silique phenotype of the *23456789* *Hyp-GALT* mutant and WT plants. The picture shows representative basal twenty-five siliques (starting from the base) on the main inflorescence stem of the *23456789* *Hyp-GALT* mutant and WT in 45-55-day-old plants grown on soil and are named S1-S25. (**B**) Quantification of mean seed count and (**C**) mean silique length of the basal twenty-five siliques S1-S25 on the main inflorescence. Data presented means ± SD (*n*=10 plants from three independent experiments). Scale bar = 1.0 cm.


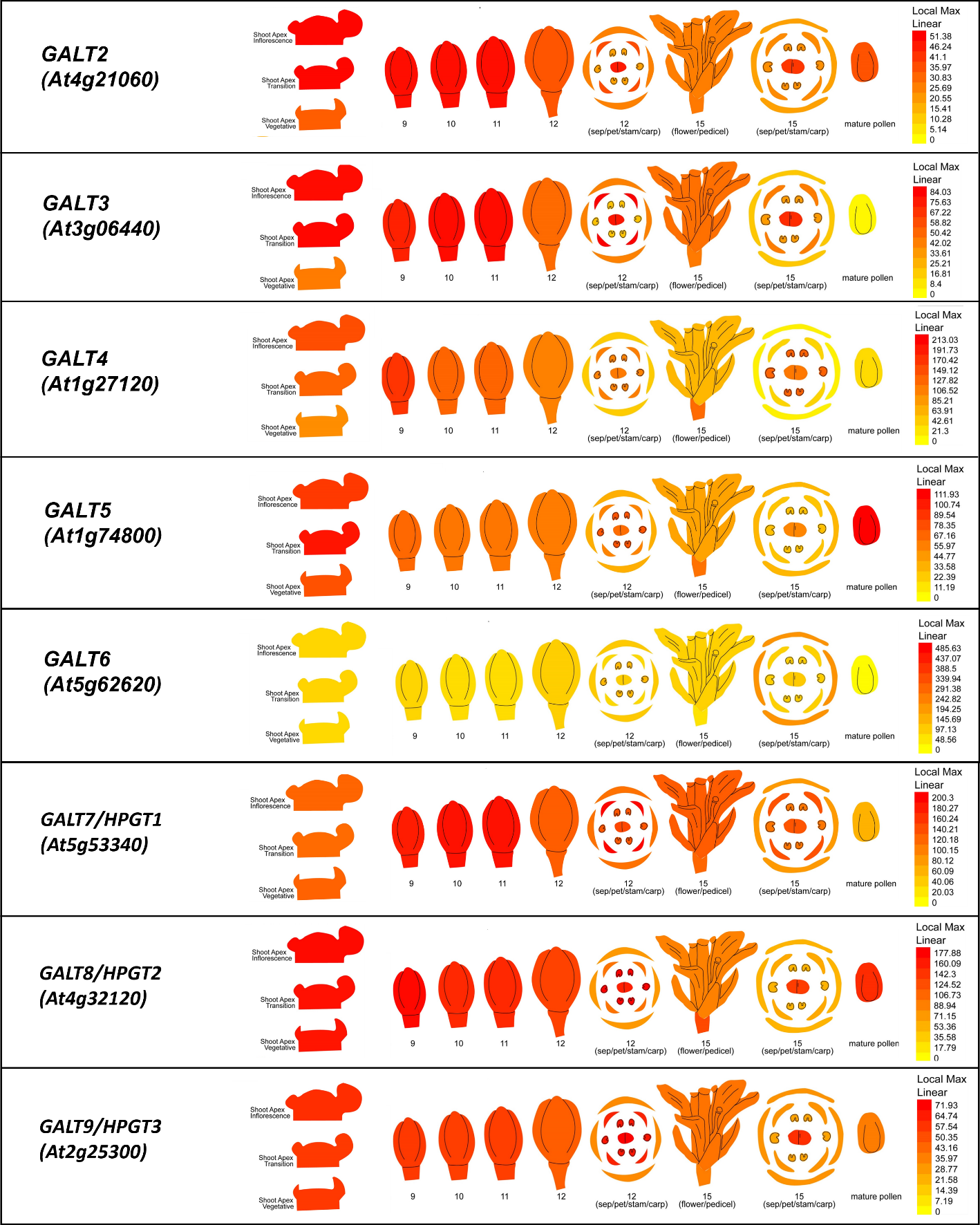
**Fig. S6.** Gene expression analysis of the eight *Hyp-GALT* genes during floral meristem and flower development. BAR ePlant browser (Waese et al., 2017) displays gene expression profiles (http://bar.utoronto.ca/eplant/).


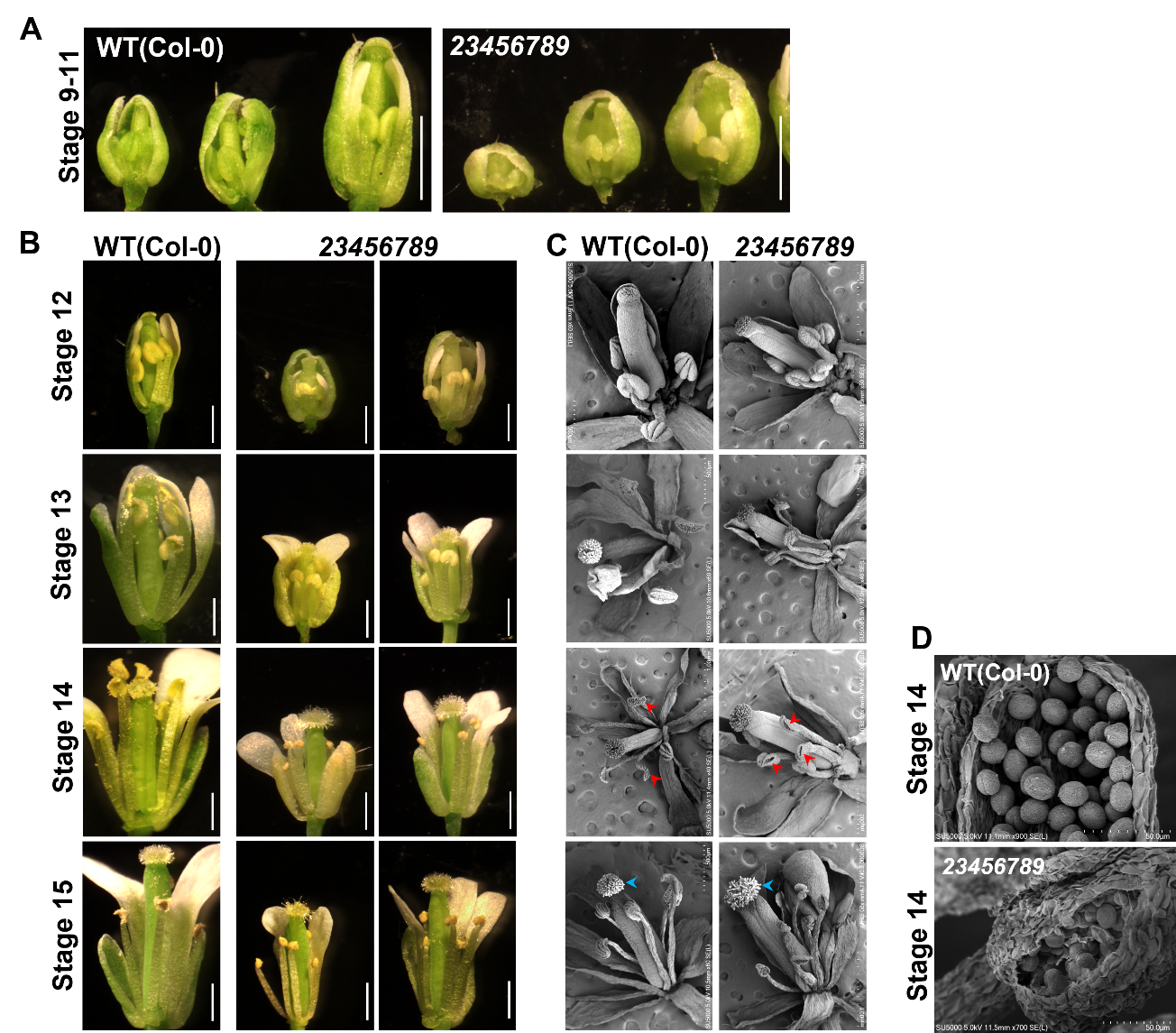
**Fig. S7. Comparative analysis of developmental stages of WT and *galt23456789* octuple mutant flowers analyzed with stereomicroscope and scanning electron micrographs.** (**A**) Flower stages floral stage 9-11 for WT and *23456789*, with the mutant displaying smaller bud sizes. (**B**) Stage 12-15 for *23456789* (both more and less severe cases) mutant shows smaller flower size with smaller partially dehiscent anthers, shorter filaments and pistils at all stages compared to the corresponding WT(Col-0) stages (**C**) SEM images of *23456789* display stigmatic area growing larger (indicated by blue arrowheads) from stage 12-15 compared to the WT due to scarcity of pollen, late and partial dehiscence along with smaller filament and anther (indicated by red arrowheads). One sepal and one petal were removed at the front in panel **A** and **B**. (**D**) Flower stage 14 of WT shows an enlarged view of completely open anthers while anther dehiscence just started in the *23456789* mutant. Scale Bars = 500 µm in (**A, B, C**), 50 µm in (**D**).


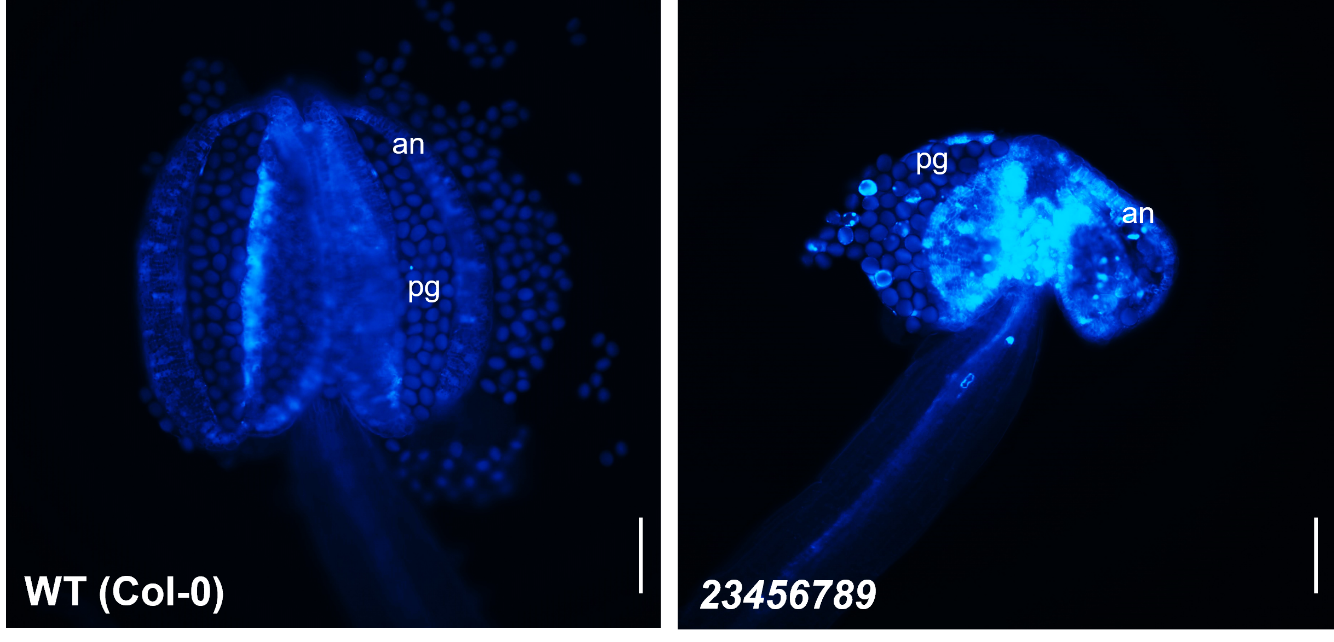
**Fig. S8. Callose staining of anthers at anthesis from the *23456789 Hyp-GALT* mutant and WT.** Pollen grains from the anther at anthesis stage were stained by aniline blue (for callose staining). Fluorescence microscopy results appeared to be intense in pollen grains as well as anthers compared to the anthesis stage in the WT. Scale bars = 50 µm. pg, pollen grains; an, anther.

**Table S1**. List of PCR primers used for confirming the *galt25789* quintuple mutants.

| **Allele** | **Primer sequence (5’ to 3’)** | | **Amplicon size (bp)** | |
| --- | --- | --- | --- | --- |
|  |  |  | **Mutant** | **Wild-type** |
| *galt2-2* | Forward | GCTTCTGCTATTGACCTGCAC | 449-749 | 997 |
|  | Reverse | ATCCTCCTTATGCCAATGGAC |  |  |
|  | LBb1.3 | ATTTTGCCGATTTCGGAAC |  |  |
| *galt5-1* | Forward | TTTCCACTTTCGACAATTTGG | 520-820 | 1197 |
|  | Reverse | CTAATTACATGGTTTTGCGGG |  |  |
|  | LBb1.3 | ATTTTGCCGATTTCGGAAC |  |  |
| *galt7-1* | Forward | TCTTTTATTGGGGTTTAGGGG | 461-761 | 1094 |
|  | Reverse | CACGGTTCTGAGAAGACTTCG |  |  |
|  | LBb1.3 | ATTTTGCCGATTTCGGAAC |  |  |
| *galt8-1* | Forward | CCCCTCTGTTAGACCGAAAAC | 499-799 | 1144 |
|  | Reverse | TTTAGGTTATGGCAAGATGCG |  |  |
|  | LBb1.3 | ATTTTGCCGATTTCGGAAC |  |  |
| *galt9-1* | Forward | AGCTTTACCTCCGACTTGTGG | 477-777 | 1052 |
|  | Reverse | TTGTCATCCACCGTAAGAACC |  |  |
|  | LBb1.3 | ATTTTGCCGATTTCGGAAC |  |  |

**Table S2.** List of primers used for sequencing four target *GALT* genes.

| **Primer name** | **gRNA target sequence** | **Primer sequence** | **Expected amplicon size** |
| --- | --- | --- | --- |
| 3-3_At3G06440_F | GTACTTCTTGAGCTTCCTTG**TGG** | GAAGAAGTGTCCAATGGAATGC | 606bp |
| 3-3_At3G06440_R |  | CGGTCTGTTTGTTGCAGAGA |  |
| 4-2_At1g27120_F | GAGAGCTTCTTCGACAATGG**CGG** | GCGGAAAATGAGGGAATTTA | 354bp |
| 4-2_At1g27120_R |  | ATCGCCGTCTTATCACCATC |  |
| 4-3_At1g27120_F | GTTCAACGAGACTAGACCAG**TGG** | TAGCTCCATTATCACCCATC | 410bp |
| 4-3_At1g27120_R |  | TTCATTGTCGATGATTTTGA |  |
| 6-3_At5G62620_F | GGCTTAGTCGATTAATAGGT**CGG** | GGCTCAGCACAAAGATGTGA | 473bp |
| 6-3_At5G62620_R |  | ACCACGCACATAGACAACCA |  |
| 6-3_At5G62620_F | GGCTTAGTCGATTAATAGGT**CGG** | CCACCTCGGATACTCCACTTGA | 832bp |
| 6-3_At5G62620_R |  | GTTTATGTTGCATCCACGACCT |  |

**Table S3.** List of primers for sequencing off-targets of the *GALT* genes.

| **Primer name** | **Off-target sequence** | **Primer sequence** |
| --- | --- | --- |
| 3-3_At2g13520_F | GAACTCTTTGAGCTTCCTTCTGG | CCAAATTGGCCTAGAATTGC |
| 3-3_At2g13520_R |  | CCTTTGCTTGAAAACCATTCA |
| 3-3_At3g46760_F | TTACTACTTGAGTTTGCTTGTGG | CAGGAACCCAATGCTAGGAA |
| 3-3_At3g46760_R |  | TTCATGGTACGGTGTTGGAA |
| 3-3_At2G10600_F | GTACTTCTTTAACTTCTTTTTAG | GAAAGAA ACTTCAGAATGCAGCCC |
| 3-3_At2G10600_R |  | GTAAAGTGGTTCTTGACCCTCT |
| 3-3_At1G55610_F | ATACTTCTTGAGCTTCTGTCTGG | CTGGACGAAGATTTCGAAGC |
| 3-3_At1G55610_R |  | GGTTACAAGCTCCGGATCAA |
| 3-3_At2G26600_F | GTTCTTCTTGGTCTTCTTTGCGG | CCGGAGATTGATCCAAGGTA |
| 3-3_At2G26600_R |  | CCAAAAACAAGCGTGGAAA |
| 4-2_At2g07070_F | GAGATAGTCTTAGACAATGGCGG | CCACCGCAGAATA ATGTTTATGA |
| 4-2_At2g07070_R |  | CTTGGATATGGAGATCTCTTCTT |
| 4-3_At2g34500_F | GTTTAGCGAGACAAGACAAGAGG | TCCACGTCATCACTCCTCTG |
| 4-3_At2g34500_R |  | TTGAAATCCAACAACGACGA |
